# Characterisation of PDGF-BB:PDGFRβ signalling pathways in human brain pericytes: evidence of disruption in Alzheimer’s disease

**DOI:** 10.1038/s42003-022-03180-8

**Published:** 2022-03-17

**Authors:** Leon C. D. Smyth, Blake Highet, Deidre Jansson, Jane Wu, Justin Rustenhoven, Miranda Aalderink, Adelie Tan, Susan Li, Rebecca Johnson, Natacha Coppieters, Renee Handley, Pritika Narayan, Malvindar K. Singh-Bains, Patrick Schweder, Clinton Turner, Edward W. Mee, Peter Heppner, Jason Correia, Thomas I.-H. Park, Maurice A. Curtis, Richard L. M. Faull, Mike Dragunow

**Affiliations:** 1 Department of Pharmacology and Clinical Pharmacology, Faculty of Medical and Health Sciences, University of Auckland, 85 Park Road, Grafton, Auckland 1023 New Zealand; 2 Centre for Brain Research, Faculty of Medical and Health Sciences, University of Auckland, Auckland, New Zealand; 3 Centre for Free Radical Research, Department of Pathology and Biomedical Science, University of Otago, Christchurch, New Zealand; 4 Center for Brain Immunology and Glia, Department of Pathology and Immunology, Washington University in St Louis, Missouri, USA; 5 Department of Anatomy with Medical Imaging, Faculty of Medical and Health Sciences, University of Auckland, 85 Park Road, Auckland, 1023 New Zealand; 6 Department of Psychiatry and Behavioural Sciences, University of Washington, Seattle, Washington USA; 7 Laboratory of Nervous System Disorders and Therapy, GIGA-Neurosciences Research Centre, University of Liège, 4000 Liège, Belgium; 8 School of Biological Sciences, University of Auckland, Auckland, New Zealand; 9 Auckland City Hospital, 2 Park Road, Auckland, 1023 New Zealand

**Keywords:** Cellular neuroscience, Preclinical research

## Abstract

Platelet-derived growth factor-BB (PDGF-BB):PDGF receptor-β (PDGFRβ) signalling in brain pericytes is critical to the development, maintenance and function of a healthy blood-brain barrier (BBB). Furthermore, BBB impairment and pericyte loss in Alzheimer’s disease (AD) is well documented. We found that PDGF-BB:PDGFRβ signalling components were altered in human AD brains, with a marked reduction in vascular *PDGFB*. We hypothesised that reduced PDGF-BB:PDGFRβ signalling in pericytes may impact on the BBB. We therefore tested the effects of PDGF-BB on primary human brain pericytes in vitro to define pathways related to BBB function. Using pharmacological inhibitors, we dissected distinct aspects of the PDGF-BB response that are controlled by extracellular signal-regulated kinase (ERK) and Akt pathways. PDGF-BB promotes the proliferation of pericytes and protection from apoptosis through ERK signalling. In contrast, PDGF-BB:PDGFRβ signalling through Akt augments pericyte-derived inflammatory secretions. It may therefore be possible to supplement PDGF-BB signalling to stabilise the cerebrovasculature in AD.

## Abstract (web summary)

Smyth et al. use tissue microarrays from Alzheimer’s disease (AD) patient brains to show that PDGF-BB:PDGFRβ signalling components are reduced in AD. They then use primary human brain pericytes to elucidate a pathway by which PDGF-BB:PDGFRβ signalling in brain pericytes is disrupted in AD, thus impairing the blood brain barrier.

## Introduction

Vascular pathology is a well-established feature of Alzheimer’s disease (AD)^1,2^, with reduced vascular density^3,4^, cerebral blood flow^5,6^ and blood–brain barrier (BBB) breakdown^7,8^. Central to vascular pathology in AD is the loss of pericytes, cells that ensheathe capillaries and maintain the BBB^2,3,9–11^. Furthermore, vascular pathology exacerbates other features of AD, including plaque load, neuronal loss and behavioural changes^9^. Most importantly, vascular changes occur prior to other pathologies, including amyloid deposition, implying a causative role in AD pathogenesis^5,7,8,12^. Therefore, vascular drug targets are a potential option for the treatment of AD.

Platelet-derived growth factors (PDGFs) are crucial to the development of the vascular system as well as the formation of the BBB in cerebral vessels^13^. In the vasculature, PDGF-BB is specifically secreted by endothelia^2,14^, while its receptor, PDGFRβ, is enriched in vascular mural cells, with the highest expression in pericytes, followed by vascular smooth muscle cells (vSMCs) and vascular-associated fibroblast-like cells^13,15^. Endothelial-derived PDGF-BB is sequestered in the basement membrane, which creates a concentration gradient that recruits mural cells to blood vessels^13,16–19^. The presence of pericytes around capillaries then leads to their stabilisation, and formation of the BBB^10,16^. Indeed, *Pdgfb* and *Pdgfrb*-deficient mice have a myriad of vascular phenotypes, including a complete lack of mural cell coverage, microaneurysm formation, haemorrhages and failure to form a BBB^13^. Even animals with partial deficiencies in PDGF-BB:PDGFRβ signalling show profound BBB leakage, pericyte deficiency and cognitive impairment^9,10,16,20^. Furthermore, pericytes, as opposed to vSMCs, are particularly sensitive to PDGFRβ depletion^21^, indicating that pericytes are exceptionally dependent on PDGFRβ. Interestingly, many vascular phenotypes related to PDGFRβ signalling deficiency also occur in AD.

In addition to its role in BBB development, PDGF-BB:PDGFRβ signalling facilitates pericyte vessel coverage and vascular stability. It is thought that alterations to PDGF-BB:PDGFRβ signalling in the adult vasculature may have more subtle consequences for pericytes and the BBB, which manifest over a longer period of time^22^. Furthermore, in models of disease, PDGF-BB treatment improves neurovascular stability in a mouse model of epilepsy by increasing pericyte number, coverage and survival^23^. On the other hand, PDGF-BB resistance in retinal pericytes causes apoptosis and leads to diabetic retinopathy^24^, and postnatal PDGFRβ inhibition results in pericyte deficiencies that cause microvascular dysfunction^25^. Together, these findings indicate that PDGFRβ signalling in pericytes may alter vascular function in later-onset diseases such as AD.

Recent studies suggest a role for vascular PDGF-BB:PDGFRβ signalling in AD pathogenesis in animal models and human neuropathology. *Pdgfrb*^+/−^ mice show stronger AD pathology, with reduced Aβ clearance and increased BBB leakage^9,26^. In AD, PDGFRβ signalling disruption resulting from the cleavage of the extracellular domain of PDGFRβ into the CSF is strongly correlated with BBB permeability, hypoperfusion and cognitive impairment^7,8,12^. This PDGFRβ shedding has been mechanistically linked to two AD-associated stressors, hypoperfusion and Aβ^27^. Furthermore, the AD-related immunogen interferon-gamma prevents pericytes from responding to PDGF-BB^28,29^. Finally, recent single-cell RNAseq findings from the brains of control and AD patients suggest that there are striking changes to endothelial cell abundance, even accounting for vascular regression, suggesting that PDGF-BB may be less abundant at the vasculature in AD^2^.

Due to the critical role of PDGF-BB:PDGFRβ signalling in maintaining pericyte coverage, and thus vascular stability, we hypothesised that impaired PDGF-BB:PDGFRβ signalling may promote vascular dysfunction in AD^30^. We, therefore, set out to evaluate if PDGF-BB:PDGFRβ signalling components were deficient in AD. We then dissected the molecular pathways activated by PDGF-BB and their functional consequences in primary human pericyte cultures, to define potential mechanisms for pericyte dysfunction in AD.

## Results

### Changes to vascular PDGF-BB:PDGFRβ signalling in AD

**Fig. 1:**
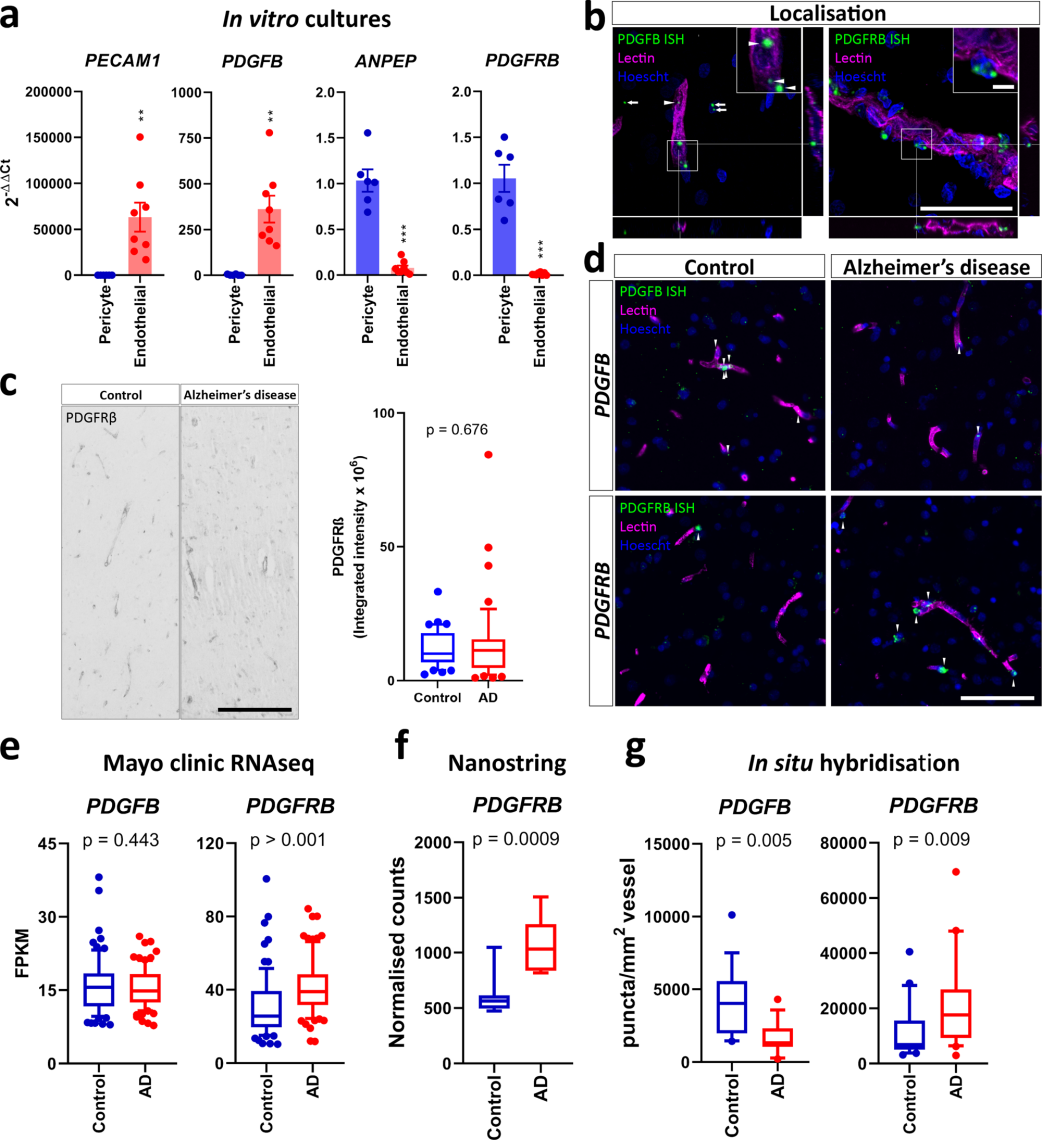
PDGF-BB:PDGFRβ signalling components are altered in the AD brain. Primary human brain pericytes and endothelial cells were isolated and PDGFRβ signalling components were measured by qPCR. **a** qPCR of lineage markers (*PECAM1* and *ANPEP*) and PDGF-BB:PDGFRβ signalling components in isolated human brain endothelial cells and pericytes. *N* = 6–8. **p < 0.01, ***p < 0.001, unpaired *t* test. Tissue microarray blocks containing control and AD cores were stained with PDGFRβ (*n* = 41–42), or UEA lectin to label blood vessels and RNAscope probes against *PDGFB* and *PDGFRB* (*n* = 14–28). **b** Confocal images of *PDGFB* and *PDGFRB* probes relative to lectin in epilepsy biopsy cortex. Scale bar = 100 μm, inset = 10 μm. **c** Representative immunohistochemical staining for PDGFRβ in control and AD cortex, and quantification of staining intensity. Scale bar = 100 μm. **d** Representative images of PDGFB and PDGFRB ISH in AD and control cortex. Scale bar = 100 μm. **e** Expression of *PDGFB* and *PDGFRB* in bulk RNAseq data of control and AD temporal cortex, derived from Allen et al. 51. *n* = 80, unpaired *t* test. **f** *PDGFRB* expression in frozen temporal cortex tissue from control and AD brains, detected by Nanostring. *n* = 9, unpaired *t* test. **g** Quantification of *PDGFB* and *PDGFRB* puncta associated with lectin-positive blood vessels. *n* = 14–21, unpaired *t* test.

Cerebrovascular pathology has been previously shown to contribute to AD^1,3^. We wished to investigate if perturbations to PDGF-BB:PDGFRβ signalling were present in AD as a potential mechanism for vascular pathology. Previous studies have indicated that, in the brain, PDGF-BB and PDGFRβ expression is confined to endothelial cells and perivascular mural cell populations, respectively^10,13,15,31^. Using isolated primary cultures and ISH, we confirmed that *PDGFB* and *PDGFRB* were specific to human brain endothelial cells and pericytes, respectively (Fig. 1a, b). We initially performed IHC against PDGFRβ, and did not observe significant changes in the abundance of the receptor in AD (Fig. 1c), however, the signal was weak so we wished to analyse changes with a more sensitive RNA-based method^32^. Bulk RNAseq of temporal cortex tissue from the Mayo Clinic late-onset AD RNAseq project revealed that there was an upregulation of *PDGFRB* in AD, but no changes in *PDGFB* (Fig. 1e)^33,34^. We also saw an upregulation of *PDGFRB* in bulk RNA from AD middle frontal gyrus in our cohort using NanoString (Fig. 1f). However, a key challenge in the interpretation of bulk RNAseq results is that they cannot discriminate between changes due to fluctuations in cell types and biological changes occurring within these cell types. We, therefore, wished to see whether these changes reflected altered pericyte and endothelial cell biology, or numbers using in situ hybridisation (ISH) in human tissue. Unlike bulk RNAseq data, which showed no change in *PDGFB* expression in the human AD brain (Fig. 1e), we observed a significant reduction in vessel-associated *PDGFB* (Fig. 1d, g). On the other hand, we observed an increase in vessel-associated *PDGFRB*, consistent with RNAseq data (Fig. 1d, g). Overall, these results suggest that PDGF-BB:PDGFRβ signalling alterations are present in AD, and may contribute to vascular pathology.

### PDGF-BB activates a biphasic response in brain pericytes

**Fig. 2:**
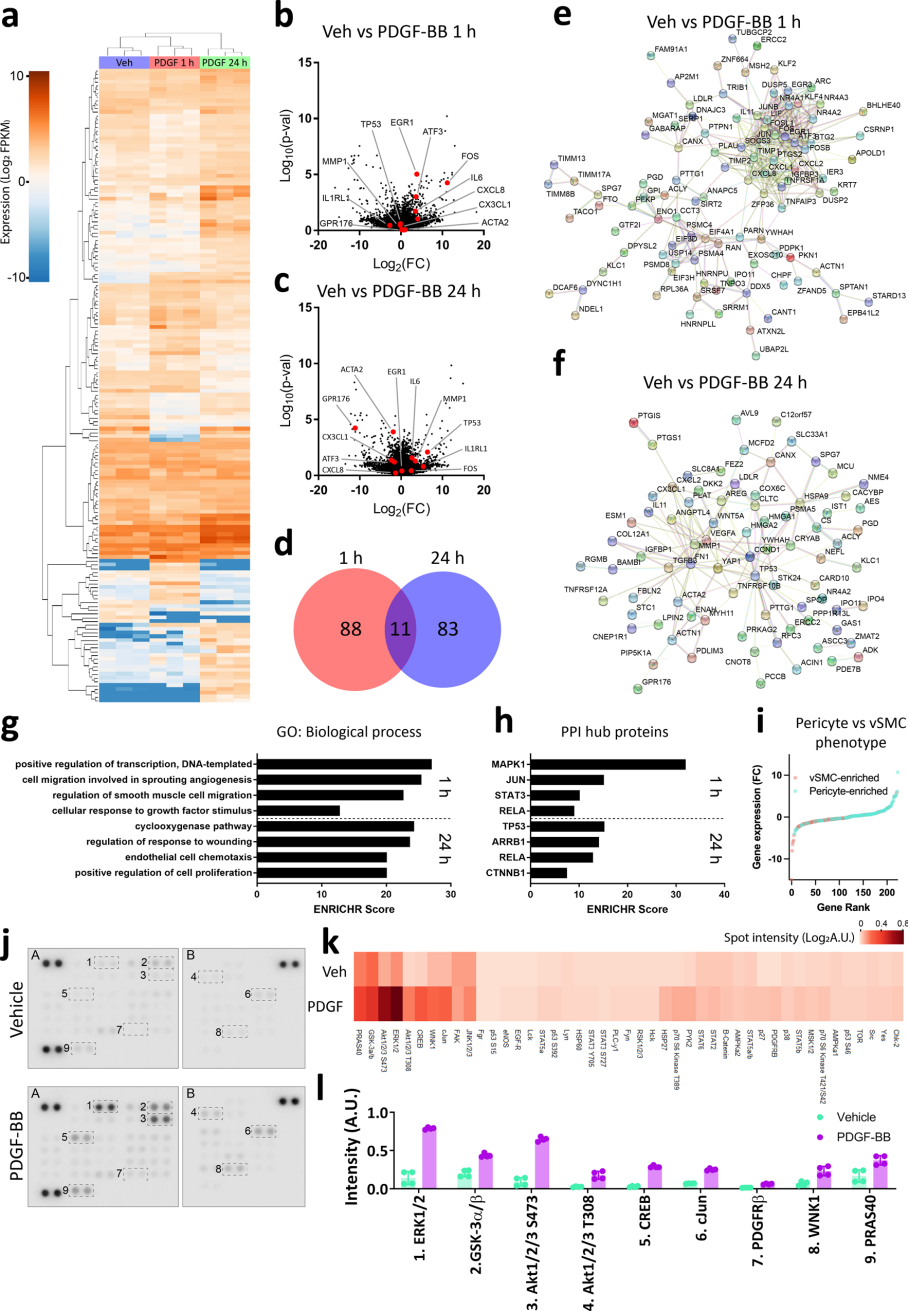
PDGF-BB activates a biphasic response in brain pericytes. Pericytes derived from neurologically normal post-mortem patients were treated with vehicle or PDGF-BB (10 ng/mL) for 1 or 24 h and gene expression analysed by RNAseq. **a** Dendrogram of PDGF-BB response in pericytes. Volcano plots comparing **b** 1 h and **c** 24 h PDGF-BB treatment to vehicle, with hits highlighted. **d** Venn diagram of differentially expressed gene lists at 1 and 24 h of PDGF-BB treatment. STRING network analysis of hits from **e** 1 and **f** 24 h of PDGF-BB treatment. **g** Gene ontology analysis and **h** interaction hub analysis of differentially expressed gene lists. **i** Expression of pericyte and vascular smooth muscle cell lineage markers identified by Vanlandewijck et al.^15^, ranked by fold change. Pericytes derived from the middle temporal gyrus of epilepsy patients were serum starved overnight, then treated with either PDGF-BB (100 ng/mL) or vehicle and lysed. Phosphorylation was detected in lysates using an antibody array. **j** Representative images, **k** heatmap of all phosphorylation sites and **l** levels of selected phosphorylation events in vehicle or PDGF-BB-treated pericytes.

We hypothesised that reduced levels of PDGF-BB secretion by brain endothelial cells may cause changes to pericytes. We wished to determine what changes may occur if PDGF-BB is less abundant so we characterised the PDGF-BB response in vitro using primary human brain pericytes. RNAseq was performed on pericytes treated with PDGF-BB for 1 or 24 h, and the transcriptome analysed. Distinct gene signatures were observed in pericytes treated with PDGF-BB for 1 and 24 h (Fig. 2a, d). Acute PDGF-BB responses included an induction of immediate early genes (IEG) *EGR1*, *KLF2*, *KLF4*, *JUN*, *FOS* and *ATF3* as well as an induction of genes encoding secretory proteins: *IL6* and *CXCL8* (Fig. 2b, e). Following 24 h of PDGF-BB treatment, IEGs induced at 1 h returned to baseline, whereas *MMP1*, *IL1RL1* and *TP53* had been induced, with *CX3CL1, ACTA2, TAGLN* and *GPR176* strongly suppressed (Fig. 2c, f). Gene ontology analysis suggested that PDGF-BB signalling regulates angiogenic and healing processes, as well as proliferation, with interaction hubs around MAPK1, RELA and JUN (Fig. 2g, h). Cross-referencing our data with pericyte and vSMC lineage transcripts identified by Vanlandewijck et al.^15^ suggested that 24 h PDGF-BB treatment causes the enrichment of pericyte transcripts and depletion of vSMC lineage markers (Fig. 2i). Furthermore, we used a meso-scale screen for phosphorylation in key intracellular pathways using an antibody array, and detected that ERK and Akt were the predominant pathways activated in pericytes, with lesser activation of GSK-3α/β and WNK1 (Fig. 2j–l).

### PDGF-BB causes the internalisation and degradation of PDGFRβ

**Fig. 3:**
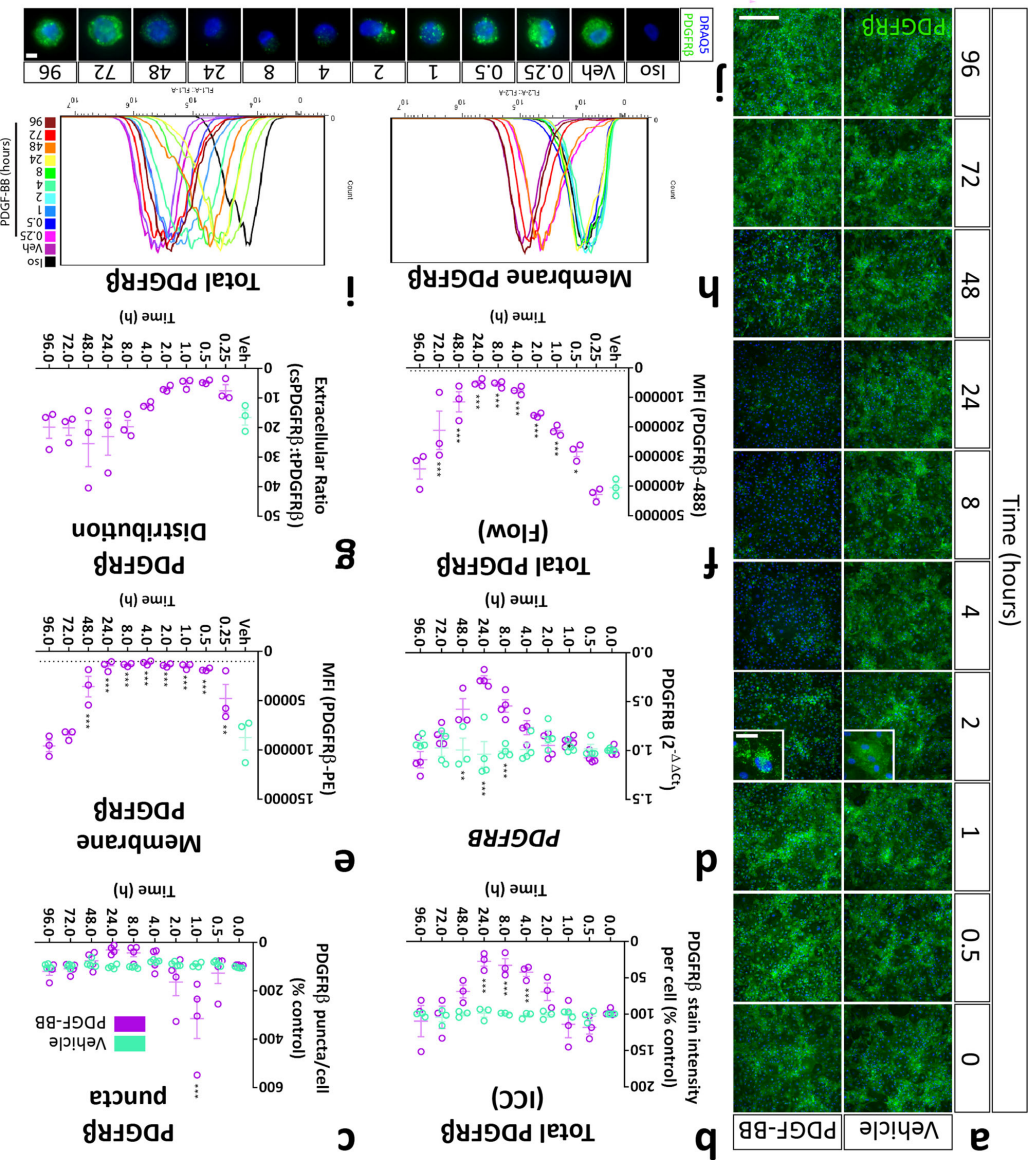
PDGF-BB treatment causes a transient loss of PDGFRβ expression due to internalisation in pericytes. Brain pericytes were treated with PDGF-BB (10 ng/mL) or vehicle for the specified time, then stained for cell-surface PDGFRβ for flow cytometry, fixed and stained for total PDGFRβ expression or RNA extracted for qPCR. **a** Representative images and quantification of **b** total PDGFRβ immunostaining and **c** PDGFRβ puncta formation in pericytes treated with vehicle or PDGF-BB. Scale bar = 500 μm, inset = 25 μm. *n* = 3–4, two-way ANOVA. **d** Gene expression of *PDGFRB* in pericytes incubated for different lengths of time with PDGF-BB. Expression of **e** cell surface and **f** total PDGFRβ and **g** the distribution of PDGFRβ determined by flow cytometry. *n* = 3, one-way ANOVA. Representative histograms of **h** cell surface and **i** total PDGFRβ and **j** representative images of total PDGFRβ expression in cells analysed by flow cytometry. Scale bar = 5 μm. *p < 0.05, **p < 0.01, ***p < 0.001 vs vehicle control, ^#^*p* < 0.05, ^##^*p* < 0.01, ^###^*p* < 0.001 vs PDGF-BB-treated.

**Fig. 4:**
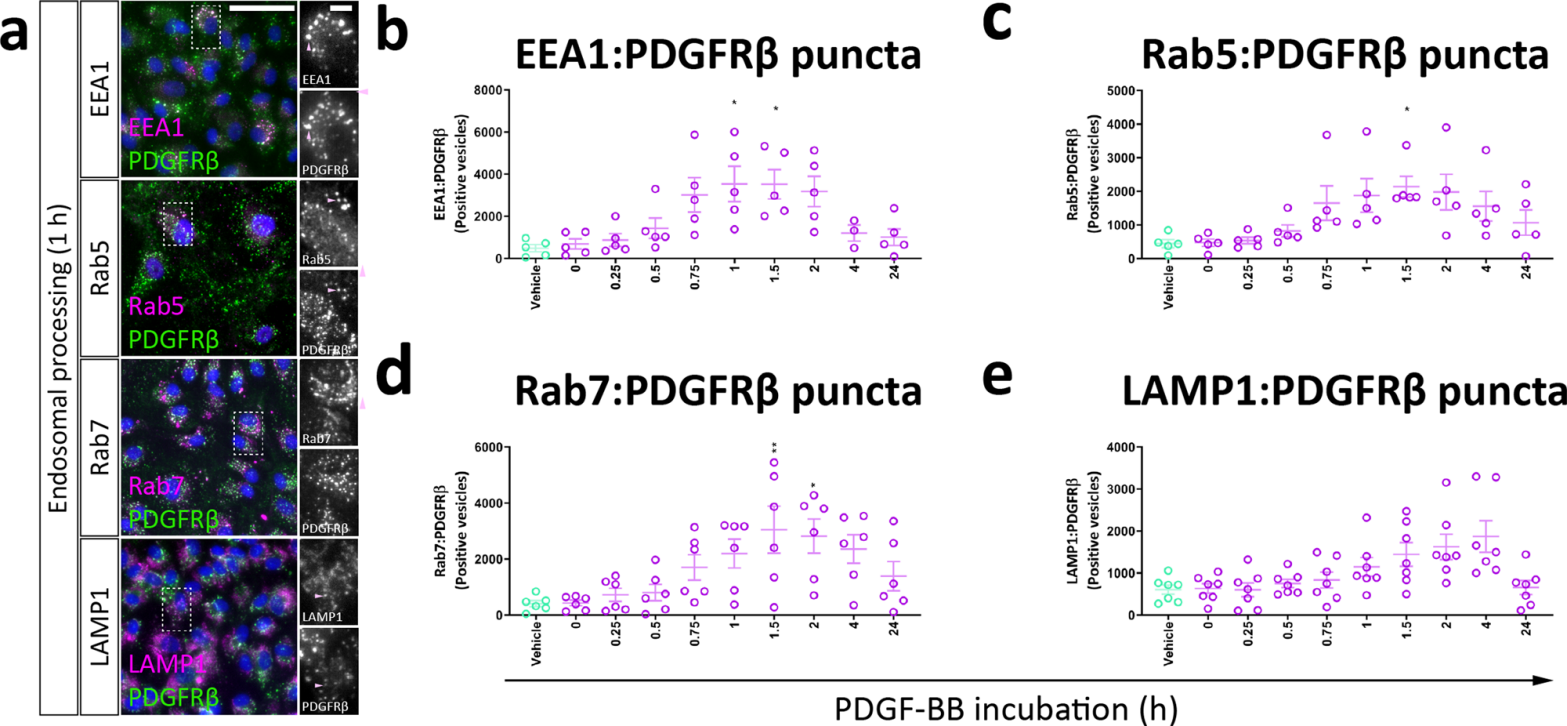
PDGF-BB causes PDGFRβ degradation through lysosomal processing in pericytes. Brain pericytes were treated with PDGF-BB (10 ng/mL) for different lengths of time, then fixed and stained for endo/lysosomal markers. **a** Representative images of PDGFRβ colabelling with EEA1, Rab5, Rab7 and LAMP1. Scale bar = 50 μm, inset = 10 μm. Quantification of PDGFRβ puncta colocalised with **b** EEA1, **c** Rab5, **d** Rab7 and **e** LAMP1. *n* = 5, one-way ANOVA. *p < 0.05, **p < 0.01, ***p < 0.001 vs vehicle control, ^#^*p* < 0.05, ^##^*p* < 0.01, ^###^*p* < 0.001 vs PDGF-BB-treated.

To determine the effect of PDGF-BB on PDGFRβ dynamics, kinetic data were gathered on PDGFRβ expression, distribution and localisation with endosomes. PDGF-BB stimulation caused rapid reorganisation of PDGFRβ, with an increase in PDGFRβ puncta formation within one hour of stimulation (Fig. 3a–c). PDGFRβ degradation was observed subsequently, being prominent between four and 24 h post stimulation, followed by later re-synthesis (Fig. 3a, c), consistent with previous reports^28^. The loss of PDGFRβ protein expression was mirrored by a reduction in *PDGFRB* gene expression, most prominent 24 h post stimulation (Fig. 3d). Flow cytometric analysis of PDGFRβ distribution mirrored immunocytochemistry, revealing rapid internalisation of the receptor within 15 min of stimulation, with subsequent loss of total PDGFRβ signal indicative of degradation (Fig. 3e–i, k). However, PDGFRβ returned to baseline membrane:intracellular distribution within eight hours of stimulation (Fig. 3g). Following internalisation of PDGFRβ, colocalisation was observed between endosomal markers and PDGFRβ puncta (Fig. 4a), with a progression from PDGFRβ:EEA1/Rab5/Rab7 positive endosomes (Fig. 4b–d) to PDGFRβ:LAMP1-positive lysosomes (Fig. 4e), indicating receptor internalisation and degradation. These data are consistent with PDGFRβ internalisation between 15 min and 2 hours post-stimulation and degradation of PDGFRβ between 4 and 8 hours post-stimulation (Fig. 3).

### Distinct aspects of the PDGF-BB response are mediated by ERK and PI3K signalling

**Fig. 5:**
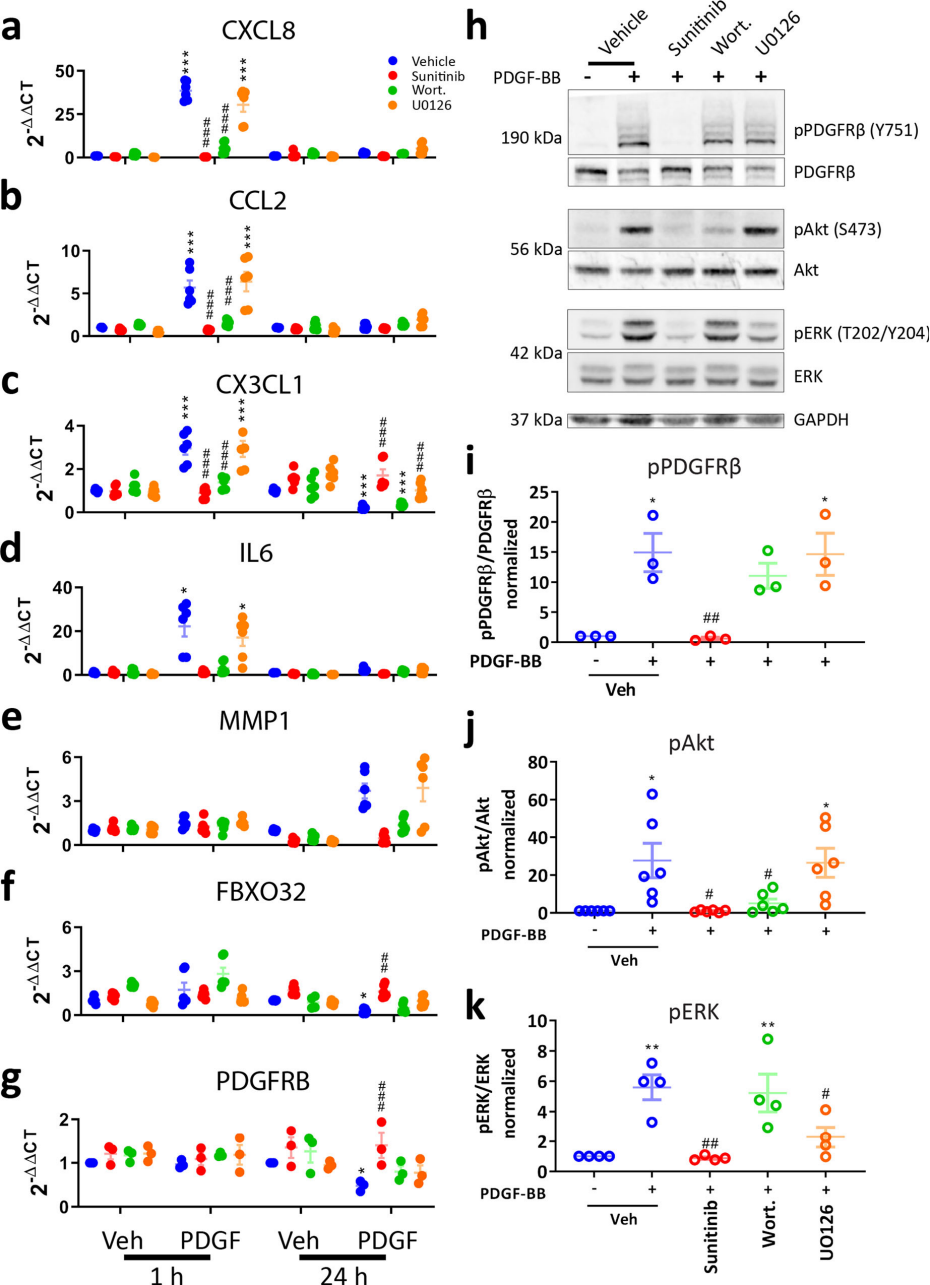
Akt and ERK activate distinct aspects of the PDGF-BB response in pericytes. Pericytes were incubated with vehicle (0.3% DMSO), PDGFRβ inhibitor sunitinib (100 nM), PI3K inhibitor wortmannin (100 nM) or MEK/ERK inhibitor U0126 (10 μM) for 30 min. Pericytes were then treated with either vehicle or PDGF-BB (10 ng/mL) for 1 or 24 h, and RNA extracted for qPCR. Expression of **a** *CXCL8*, **b** *CCL2*, **c** *CX3CL1*, **d** *IL6*, **e** *MMP1*, **f** *FBXO32* and **g** *PDGFRB* in pericytes treated with PDGF-BB for 1 or 24 h, with or without pathway inhibitors. *n* = 6, two-way ANOVA. Pericytes were serum starved overnight, then pre-treated with sunitinib (100 nM), wortmannin (100 nM) or U0126 (10 μM) or vehicle (0.3% DMSO) for 30 min, then treated with vehicle or PDGF-BB (100 ng/mL) for 30 min. Cells were lysed and western blot was performed. **h** Representative blots and densitometric analysis of **i** PDGFRβ, **j** Akt and **k** ERK phosphorylation. *n* = 4, one-way ANOVA. *p < 0.05, **p < 0.01, ***p < 0.001 vs vehicle control, ^#^*p* < 0.05, ^##^*p* < 0.01, ^###^*p* < 0.001 vs PDGF-BB-treated.

**Fig. 6:**
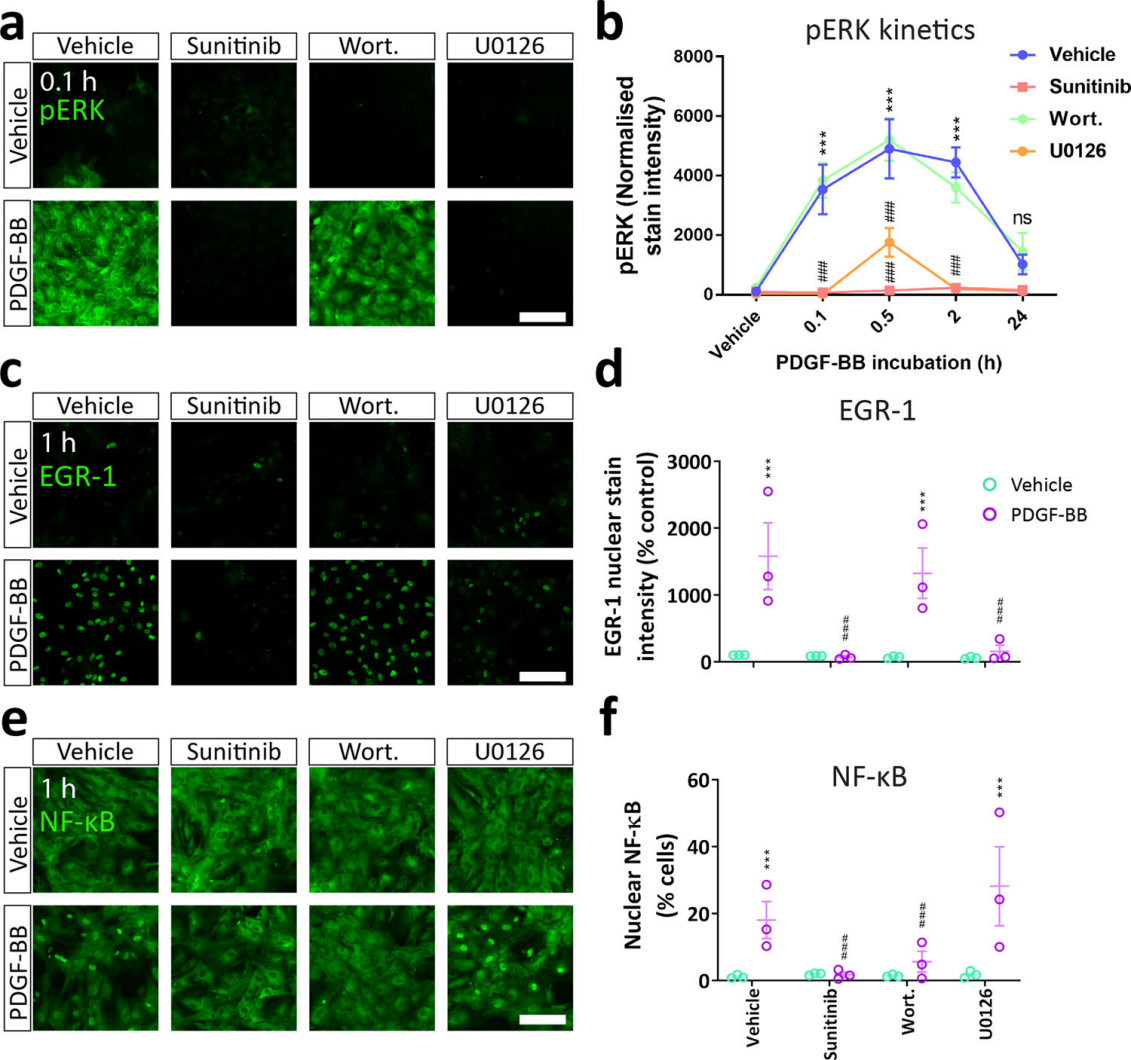
PDGF-BB activates Akt-dependent NF-κB and ERK-dependent EGR-1 signalling in pericytes. Pericytes were pre-treated with sunitinib (100 nM), wortmannin (100 nM) or U0126 (10 μM) or vehicle (0.3% DMSO) for 30 min, then treated with vehicle or PDGF-BB (10 ng/mL) for the specified timepoints. Cells were fixed at endpoint and immunostained. Representative images and quantification of **a**–**b** pERK, **c**–**d** EGR-1 and **e**–**f** NF-κB activation following PDGF-BB treatment with or without pathway inhibitors. *n* = 3, two-way ANOVA. Scale bar = 100 μm. *p < 0.05, **p < 0.01, ***p < 0.001 vs vehicle control, ^#^*p* < 0.05, ^##^*p* < 0.01, ^###^*p* < 0.001 vs PDGF-BB-treated.

Because we determined that PDGF-BB robustly activates ERK and PI3K in pericytes (Fig. 2j–l), we wished to determine how these pathways governed the expression of genes augmented by PDGF-BB treatment. Because PDGF-BB is a promiscuous ligand, we determined that the effects of PDGF-BB on pericytes were on-target at PDGFRβ using small interfering RNA (siRNA) and that these effects were PDGF-BB concentration-sensitive (Supplementary Fig. 1). We wished to dissect the contribution of the ERK and PI3K pathways to the PDGF-BB response in pericytes, as well as blocking PDGFRβ to confirm that effects were PDGFRβ-dependent. We, therefore, used inhibitors of PDGFRβ (sunitinib), PI3K (wortmannin) and MEK/ERK (U0126) to examine which pathways controlled PDGF-BB-induced gene expression in the early (1 h) and late (24 h) phases of the response. We determined that the early induction of *CXCL8*, *CCL2*, *CX3CL1* and *IL6*, as well as the late induction of *MMP1* were blocked by wortmannin and therefore dependent on PI3K signalling (Fig. 5a–g). In contrast, we only found that the suppression of *CX3CL1* at 24 h was dependent on ERK (Fig. 5c), while no clear pathways were defined for the late suppression of *FBXO32* or *PDGFRB* (Fig. 5f, g). We then validated the efficacy and specificity of the pharmacological inhibitors using western blot against total and phosphorylated PDGFRβ, ERK and Akt (Fig. 5h–k). Drugs had no off-target toxicity at concentrations tested (Supplementary Fig. 2). We validated the effect of sunitinib and U0126 for the blockade of PDGF-BB-induced ERK phosphorylation (Fig. 6a, b), and also found the reduction of EGR-1 at the protein level (Fig. 6c, d). Owing to pathway analysis indicating a role for NF-κB in the PDGF-BB response in pericytes (Fig. 2h), we also examined PDGF-BB-dependent NF-κB activation. We found a subtle induction of NF-κB during the early phase of the PDGF-BB response, and determined that this effect was PI3K-dependent (Fig. 6e, f). Investigation of PDGF-BB-induced transcription factor activation revealed that both ERK and Akt are required for full activation of ATF3 and c-Jun, although we did not observe activation of STAT1, STAT3 or SMAD2/3 in pericytes as has been reported in other cell types (Supplementary Fig. 3)^35–40^.

### PDGF-BB signalling through ERK regulates pericyte proliferation and protection from apoptosis

**Fig. 7:**
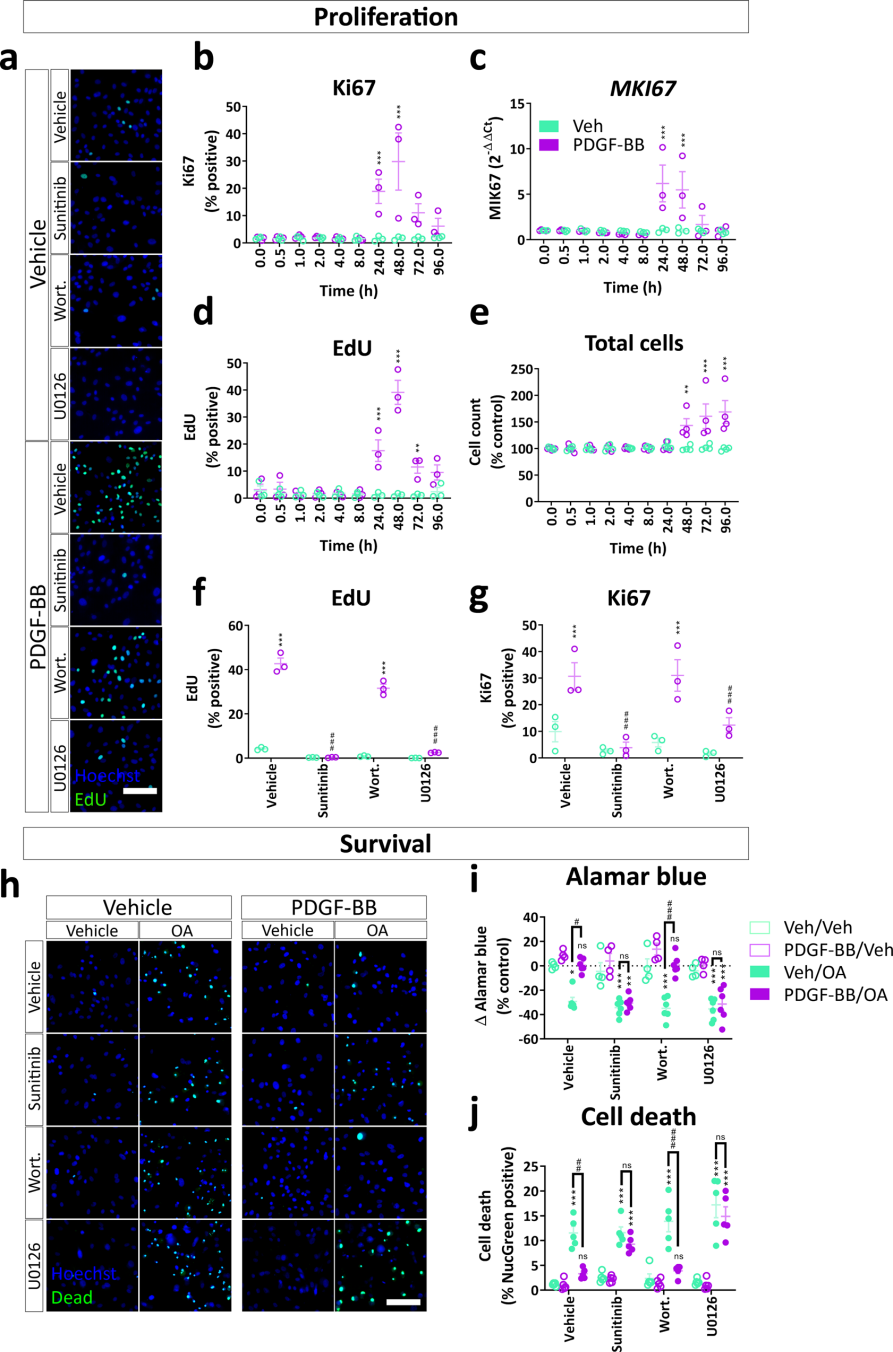
PDGF-BB-dependent proliferation and protection from apoptosis are dependent on ERK signalling. Pericytes were grown to confluence, then treated with PDGF-BB (10 ng/mL) for up to 96 h, then fixed at the endpoint and immunostained or RNA harvested for qPCR. EdU was added 24 h prior to the endpoint. Quantification of **b** Ki67 protein and **c** *MKI67* gene expression following PDGF-BB stimulation. Quantification of **d** EdU incorporation and **e** cell count following PDGF-BB treatment. *n* = 3–4, two-way ANOVA. Pericytes were pre-treated with PDGFRβ inhibitor sunitinib (100 nM), PI3K inhibitor wortmannin (100 nM) or MEK/ERK inhibitor U0126 (10 μM) or vehicle (0.3% DMSO) for 30 min, then treated with vehicle or PDGF-BB (10 ng/mL) for 48 h. EdU was added 24 h prior to endpoint. **a** Representative images and quantification of **f** EdU incorporation and **g** Ki67 immunostaining in pericytes treated with PDGF-BB with or without pathway inhibitors. Pericytes were pre-treated with sunitinib (100 nM), wortmannin (100 nM) or U0126 (10 μM) or vehicle (0.3% DMSO) for 30 min, then treated with vehicle or PDGF-BB (10 ng/mL) for 24 h. *n* = 3, two-way ANOVA. Apoptosis inducer okadaic acid (OA; 50 nM) or vehicle was added to pericytes for a further 24 h, and viability was analysed by the AlamarBlue assay or ReadyProbes™ Cell Viability Imaging Kit. **h** Representative images of viability staining of pericytes treated with PDGF-BB, pathway inhibitors and OA. Scale bar = 100 μm. **i** Quantification of AlamarBlue fluorescence and **j** viability staining in pericytes. *n* = 6, two-way ANOVA. *p < 0.05, **p < 0.01, ***p < 0.001 vs vehicle control, ^#^*p* < 0.05, ^##^*p* < 0.01, ^###^*p* < 0.001 vs PDGF-BB-treated.

PDGF-BB signalling increases pericyte proliferation in vivo and is essential for pericyte survival during development and injury^41^, therefore, we investigated the pathways mediating these responses. PDGF-BB induced a strong proliferative response in pericytes, demonstrated by an increase in Ki67 and EdU positive nuclei and *MKI67* gene expression (Fig. 7a–d). The mitogenic effect of PDGF-BB was also reflected by an increased number of total cells from 48 h onwards (Fig. 7e) that was PDGFRβ-dependent (Supplementary Fig. 1e, h, k). PDGF-BB-induced proliferation was blocked by sunitinib, and U0126, but not wortmannin, indicating that this proliferation is controlled by PDGFRβ signalling through ERK (Fig. 7f). We also determined that PDGF-BB mediates pericyte migration, independent of proliferation, however, found no evidence that ERK or Akt mediated this response (Supplementary Fig. 4). Previous studies have shown that PDGF-BB is important for pericyte survival^42,43^, and we, therefore, investigated the role of the MEK/ERK and PI3K pathways in pericyte protection. Using a nuclear inclusion dye assay (live/dead) and the AlamarBlue assay to measure viability, we determined that PDGF-BB protected pericytes from okadaic acid (OA)-induced apoptosis (Fig. 7h–j). Further, PDGF-BB-mediated protection was blocked by sunitinib and U0126, but not wortmannin (Fig. 7h–j). These data indicate that PDGF-BB acts through the ERK pathway to promote pericyte survival in stressful conditions. We found no evidence that the ERK-regulated transcription factor EGR-1 mediated any PDGF-BB-mediated effects observed in this study (Supplementary Fig. 5).

### PDGF-BB augments the secretory profile of brain pericytes through PI3K-NF-κB activation

**Fig. 8:**
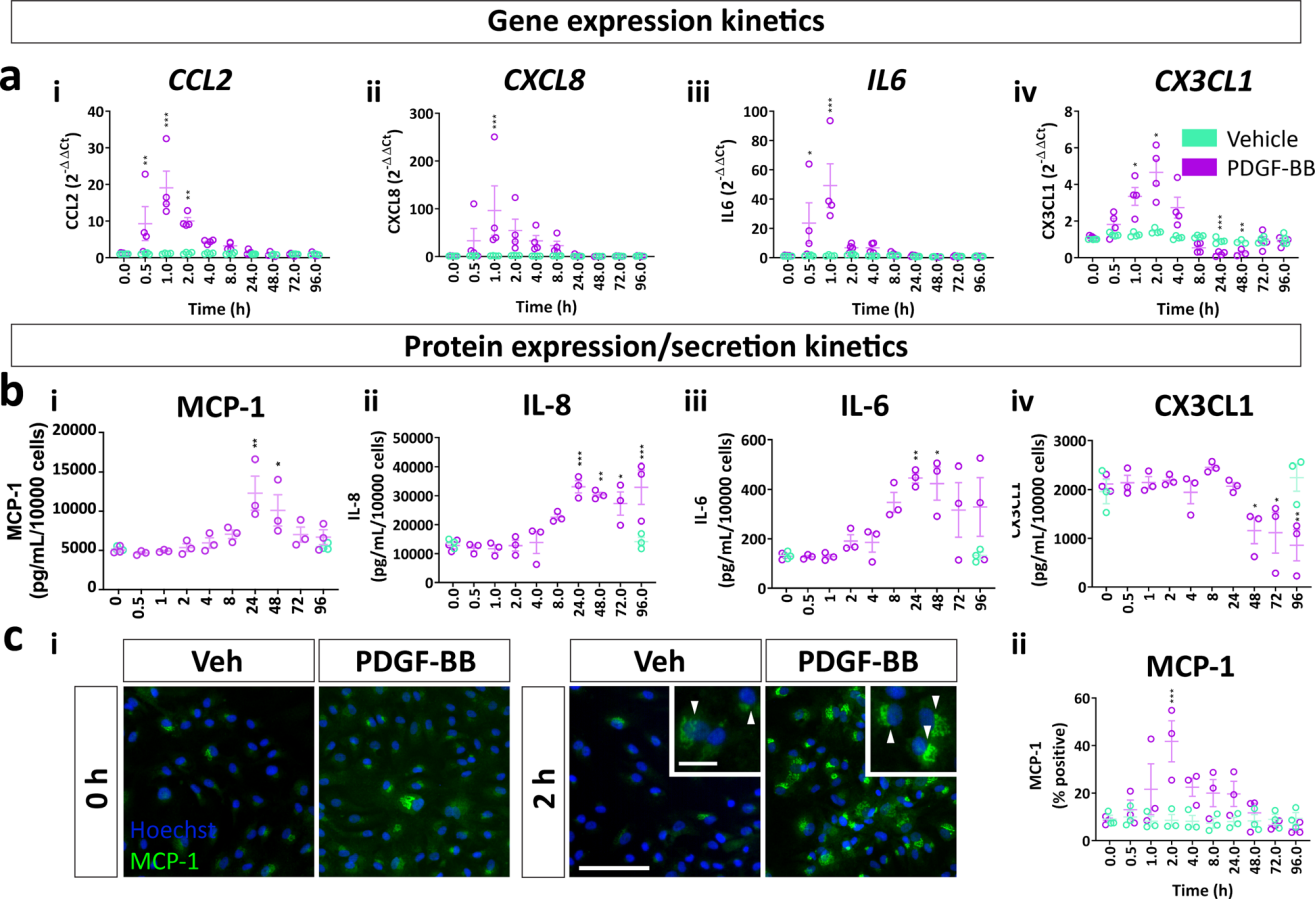
PDGF-BB alters pericyte inflammatory secretions. Pericytes were treated with PDGF-BB (10 ng/mL) or vehicle for up to 96 h, RNA extracted at endpoint and qPCR performed. **a** Kinetics of *CCL2, CXCL8, IL6* and *CX3CL1* induced by PDGF-BB expression. *n* = 4, two-way ANOVA. Pericytes were treated with PDGF-BB (10 ng/mL) or vehicle for up to 96 h, then fixed at endpoint and conditioned media collected. Samples were then immunostained and secretions in conditioned media analysed by cytometric bead array. **b** Quantification of MCP-1, IL-8, IL-6 and CX3CL1 secretion and **c** MCP-1 immunostaining in pericytes treated with PDGF-BB. *n* = 3, two-way ANOVA. Scale bar = 100 μm, insert = 10 μm. *p < 0.05, **p < 0.01, ***p < 0.001 vs vehicle control, ^#^*p* < 0.05, ^##^*p* < 0.01, ^###^*p* < 0.001 vs PDGF-BB-treated.

**Fig. 9:**
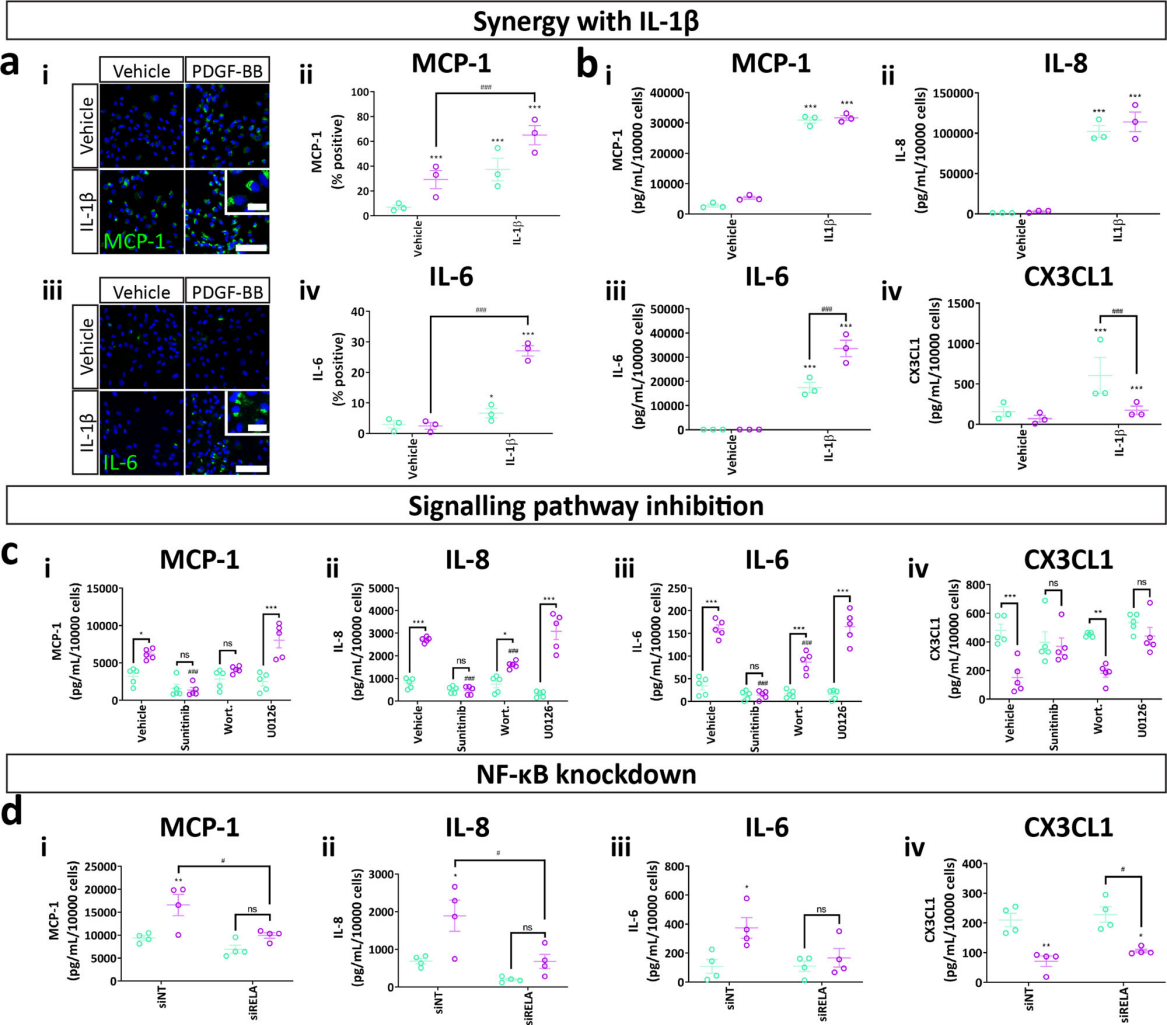
PDGF-BB acts through the PI3K-NF-κB pathway to modify pericyte inflammatory secretions. Pericytes were treated with vehicle or IL-1β (10 ng/mL), with or without PDGF-BB (10 ng/mL) for 2 h, then fixed and immunostained or 48 h and conditioned media collected for cytometric bead array. **a** Representative images and quantification of MCP-1 and IL-6 in cells treated with IL-1β or PDGF-BB. *n* = 3, two-way ANOVA. Scale bar = 100 μm, insert = 10 μm. **b** Secretion of MCP-1, IL-8, IL-6 and CX3CL1 in cells treated with IL-1β with or without PDGF-BB. Pericytes were pre-treated with PDGFRβ inhibitor sunitinib (100 nM), PI3K inhibitor wortmannin (100 nM) or MEK/ERK inhibitor U0126 (10 μM) or vehicle (0.3% DMSO) for 30 min, then treated with vehicle or PDGF-BB (10 ng/mL) for 48 h, and conditioned media collected. **c** Quantification of MCP-1, IL-8, IL-6 and CX3CL1 concentrations in conditioned media from pericytes treated with PDGF-BB with or without pathway inhibitors. Pericytes were treated with control siRNA (siNT) or siRNA directed against p65 NF-κB (siRELA) for 96 h, then treated with PDGF-BB (10 ng/mL) for 48 h and conditioned media collected for cytometric bead array. *n* = 5, two-way ANOVA. **d** Quantification of MCP-1, IL-8, IL-6 and CX3CL1 concentrations in conditioned media from pericytes treated with PDGF-BB with siRNA against NF-κB. *n* = 4, two-way ANOVA. *p < 0.05, **p < 0.01, ***p < 0.001 vs vehicle control, ^#^*p* < 0.05, ^##^*p* < 0.01, ^###^*p* < 0.001 vs PDGF-BB-treated.

Because RNAseq revealed the induction of genes encoding important inflammation-related secretions in the early phase of the PDGF-BB response (Fig. 2), we examined the kinetics of PDGF-BB-induced secretions. Results obtained by qPCR demonstrated that expression of *CCL2, CXCL8, IL6* and *CX3CL1* were maximally induced 1 hour post-stimulation, with CX3CL1 also strongly suppressed 24 h post stimulation (Fig. 8a). Immunocytochemistry and cytometric bead array revealed that changes in gene expression were followed by corresponding changes in protein expression with increased MCP-1 staining peaking 2 hours post stimulation (Fig. 8c), and increased MCP-1, IL-8 and IL-6 secretion all detected within 8 hours of treatment (Fig. 8b). CX3CL1 secretion was suppressed following at least 48 h of PDGF-BB treatment (Fig. 8b). Because PDGF-BB augmented the expression of several important pericyte secretions, we also examined if PDGF-BB modulated the response to the classical inflammatory mediator IL-1β. Immunocytochemistry indicated that PDGF-BB had a synergistic effect with IL-1β to increase the expression of MCP-1 and IL-6 (Fig. 9a), and synergy was observed with IL-6 at the secretion level, the same synergy could not be detected in MCP-1 secretion (Fig. 9b). In addition, PDGF-BB suppressed CX3CL1 secretion to baseline levels, even when induced by IL-1β (Fig. 9b). To dissect the contribution of ERK and Akt to PDGF-BB-modified secretions, we used pharmacological inhibitors, and found that the induction of MCP-1, IL-8 and IL-6 was dependent on Akt, whereas suppression of CX3CL1 was dependent on ERK (Fig. 9c). Furthermore, we found that knockdown of NF-κB p65 blunted the induction of MCP-1, IL-6 and IL-8, but did not alter CX3CL1 suppression (Fig. 9d, Supplementary Fig. 6).

## Discussion

PDGF-BB:PDGFRβ signalling is necessary to pericyte investment and vascular stability in development^10,13,16^, and it is thought to be involved in BBB maintenance during adulthood^22^. Intriguingly, vascular pathology occurs early in AD, and is similar to that seen in pericyte-deficient animals^44^. We, therefore, characterised the potential contribution of vascular PDGF-BB:PDGFRβ signalling to vascular changes in AD. We found that PDGF-BB:PDGFRβ signalling components were modified in the AD vasculature, with a pronounced loss of endothelial *PDGFB* expression. We then performed RNAseq and functional characterisation of the PDGF-BB response in pericytes to identify pathways that may be beneficial in restoring vascular integrity in AD. Taken together, we believe that vascular PDGFRβ signalling is a potential drug target in AD.

There is a large body of evidence indicating that the neurovasculature is affected early in the development of AD^45^. Indeed, we observed a similar reduction in vascular density in the AD brain to other studies^3^. We demonstrate that *PDGFB* is still present in adult brain endothelial cells, indicating that constitutive PDGF-BB:PDGFRβ signalling plays a homoeostatic role, in addition to its known role in vascular development^10,13,16^. We observed that there was a reduction in vascular *PDGFB* puncta in AD, overcoming the limitations of conventional bulk RNAseq, which did not show a difference and is likely affected by shifts in cell populations^46^. Interestingly, in aging, brain endothelial *PDGFB* expression is rescued following inhibition of vascular alkaline phosphatase, leading to a restoration of normal BBB function^47^. Recent work also suggests that there may be heterogeneous pericyte populations in the human brain, with one subtype selectively lost in AD^2^. It will be important to determine whether there is a relationship between loss of PDGF-BB signalling and loss of this pericyte subtype in particular. Regional heterogeneity in pericytes has recently been described, and it is possible that this may be another cause of heterogeneous pericyte death and BBB leakage in AD^48^. Recent single-cell analysis of endothelial cells in the AD brain suggests that this reduction in *PDGFB* may be driven by reduced endothelial cell abundance in AD, even when accounting for vessel density, meaning that endothelial cells may not be capable of supporting the same density of mural cells as healthy vessels^2^. However, because no difference in *PDGFB* was observed in bulk RNAseq data, this suggests that non-vascular expression of *PDGFB* increases. Accordingly, recent single nuclear RNAseq of human vasculature revealed a large increase in *PDGFB* in microglia in AD^2^ . This suggests that the local vascular concentration of PDGF-BB around the vasculature is disrupted in AD. Interestingly, disruption of the local vascular concentration of PDGF-BB with deletion of its retention motif leads to pericyte deficiency in development independently of overall PDGF-BB levels^43^. Global PDGF-BB levels are therefore not sufficient for pericyte maintenance, and loss of vascular PDGF-BB expression in particular, as we observe here, is particularly detrimental. We also found that *PDGFRB* expression increases in AD, aligning with bulk RNAseq data. It is possible that the misalignment between PDGFRβ RNA and protein expression is due to fluctuations in cell populations due to neuronal death, pericyte regression^3,9^ or the post-translational shedding of the extracellular domain of PDGFRβ^7,12,27^. Previous work also suggests that the AD-related immunogen interferon-gamma induces PDGFRβ expression in pericytes, but blocks its re-synthesis^28^. It is possible that a similar inflammatory mechanism in the AD brain underlies the difference between PDGFRβ gene and protein expression. Other studies have also indicated that increased PDGFRβ expression in pericytes is a marker for their activation in stroke and traumatic brain injury^41,48–50^. Our data also support the notion that pericytes are activated in AD, showing increased *PDGFRB* expression both overall and on a per-cell level.

We also examined the pathways and functions activated by PDGF-BB in brain pericytes to interrogate vascular PDGFRβ signalling at a molecular level using in vitro cultures. Using RNAseq, we observed that PDGF-BB rapidly activates IEGs, which are important in governing PDGF-BB signalling specificity in vivo^51^. Indeed, we saw the induction of *KLF2* and *KLF4* leading to the reduced expression of vSMC phenotype markers (*ACTA2*, *TAGLN*, *MYH11*) later on, which has been documented in pericytes previously^52–54^. We identified that PDGFRβ-dependent ERK activation promoted pericyte proliferation and survival in stressful conditions. We hypothesise that, in a toxic AD environment^9,26,27^ and without PDGF-BB to prevent cell death and encourage proliferation^22^, pericytes cannot turn over and are more likely to die, although it will be important to describe PDGF-BB-dependent protection in an in vivo setting. We, therefore, expect that PDGFRβ agonists may protect pericytes during AD pathogenesis, particularly if they can activate ERK signalling.

Consistent with previous studies, we observed profound activation of the PI3K pathway^28^, and PI3K-dependent NF-κB activation by PDGFRβ signalling^55^. RNAseq also revealed that PDGF-BB augmented genes encoding major pericyte secretions (*CCL2, CXCL8, CX3CL1, IL6*)^56,57^. We were able to correlate the induction of *CCL2*, *CXCL8* and *IL6* with PI3K-induced NF-κB signalling, while *CX3CL1* suppression was ERK-dependent. Interestingly, the PI3K inhibitor wortmannin was not able to block PDGF-BB-mediated protection as others have reported^55^, indicating that human brain pericyte PDGF-BB signalling is functionally distinct from related cells like fibroblasts, potentially due to differences in IEG induction^51^. Although the level of secretions induced by PDGF-BB is low compared to IL-1β, we hypothesise that these low levels of secretions may provide local trophic support to endothelial cells, without activating nearby immune cells. This notion is supported by the observation that low levels of endothelial inflammatory activation promote angiogenesis in the presence of pericytes^58^. We also expect that there may be secretions that were induced by PDGF-BB, but were not pursued here (*HBEGF, TGFB3, FGF2*, *LIF*, *IL11*, *ANGPTL4*), that are important to regulating pericyte-endothelial communication and vascular integrity^59,60^. In the context of AD, reduced PDGFRβ signalling in pericytes may reduce the constitutive secretion of these trophic factors, sensitising endothelial cells to damage by AD-related stressors. On the other hand, our observation that PDGF-BB sensitises pericytes to IL-1β indicates that high levels of PDGF-BB may be detrimental in the context of acute inflammation. In particular, it is possible that enhanced pericyte-derived MCP-1 may perpetuate glial inflammation, as has been found in sepsis and seizures^61,62^. It is important to note that the secretions in these cultures may be different from pericytes in vivo, and is a key limitation of in vitro work. However, it is notable that brain pericyte inflammatory response to an in vivo LPS challenge mirrors in vitro changes observed in our cultures^63^.

Here, we identified molecular and functional consequences of PDGFRβ signalling in primary human brain pericytes for the first time. The majority of this work uses in vitro models, and it will be important to determine the functional consequences of the exposure to PDGF-BB are also relevant in vivo. This report also provides evidence for impaired PDGF-BB:PDGFRβ signalling in AD, and identifies potential pathways that may be lost in pericytes in the AD brain. This raises the possibility that PDGFRβ agonists may also be used to supplement deficient PDGF-BB levels in AD to improve vascular function.

## Methods

### Immunohistochemistry and ISH

Tissue from the middle temporal gyrus of neurologically normal (control), and clinically and pathologically confirmed AD cases were processed to generate formalin-fixed paraffin-embedded (FFPE) blocks, as described previously^64,65^. Tissue microarray (TMA) blocks containing cores from the middle temporal gyrus of control (*n* = 18–28) and AD (*n* = 14–21) brains were generated as described previously (See Supplementary Table 1 for details)^65,66^. Sections (7 µm) were then cut on a rotary microtome (Leica Biosystems RM2235) and mounted onto slides prior to labelling. RNAscope-based ISH was performed against the human *PDGFB* and *PDGFRB* genes, with the RNAscope® 2.0 HD Detection Kit (FastRed) as described in the manufacturer’s instructions (Probes: Hs-PDGFB, Cat No. 406701; Hs-PDGFRB, Cat No. 548991; ACDBio). FFPE-embedded epilepsy-derived sections (*n* = 1) were used as positive controls for *PDGFB* and *PDGFRB* expression (Fig. 1c; see Supplementary Table 1 for details). Following ISH, antibody labelling was performed as described previously^64^, and sections were coverslipped with ProLong diamond antifade reagent (Invitrogen; P36965). Slides were imaged with a ×10 objective using a Zeiss Axio Imager Z2 (Carl Zeiss, Germany) VSlide scanner (MetaSystems, Version 2.1.124). Antibody details are provided in Supplementary Table 4. Representative images of the localisation of *PDGFB* and *PDGFRB* puncta were taken on an Olympus FV1000 confocal microscope (×63 objective, NA 0.95; Biomedical Imaging Research Unit, University of Auckland).

### Image analysis for tissue staining

FastRed-positive puncta were counted using a custom ImageJ module as described previously^32^. Endothelial expression of *PDGFB* was quantified by detecting puncta within a lectin mask, whereas pericyte expression of *PDGFRB* was quantified within a dilated lectin mask. Puncta counts were then normalised to lectin-positive area. To confirm that differences in *PDGFB* and *PDGFRB* puncta signals were not due to differences in post-mortem delay or age at death, we ran regressions but no significant associations were detected between either factor and either gene (Supplementary Fig. 7, see Supplementary Table 8 for details).

### Primary human pericyte cultures

Pericytes were derived from the middle temporal gyrus of tissue resected during anterior temporal lobectomy surgeries for the treatment of drug-refractive epilepsy or autopsy specimens, as described previously (See Supplementary Table 2 for details)^31,67^. All specimens were collected with written patient consent, and all protocols were approved by the Northern Regional Ethics Committee (New Zealand). In brief, tissue was mechanically dissociated using scalpels, enzymatically dissociated (Papain (0.25 U/g tissue; Worthington, NJ, USA), DNase I (10 U/g tissue; Invitrogen, CA, USA), 30 min at 37 °C with agitation), and the resulting suspension passed through a cell strainer (100 µm). The suspension was grown in Dulbecco’s modified Eagle medium:F12 supplement (DMEM:F12; Gibco, CA, USA) supplemented with fetal bovine serum (10%, FBS; Gibco), GlutaMAX (1%; Gibco) and penicillin/streptomycin (50 U/mL penicillin, 50 µg/mL streptomycin; Gibco), henceforth DMEM. Owing to the proliferation of pericytes, and not the other cell types that are present at early passage, serial passaging (more than passages) yields pericyte monocultures, as we have shown previously^67^. Pericytes were grown in DMEM until confluent, then dissociated by trypsinisation (0.25% trypsin, 1 mM ethylenediamine tetraacetic acid; Gibco), and seeded at a density of 15,000 cells/cm^2^ for experimentation. For transcriptome analysis and analysis of disease phenotype, pericytes from the middle temporal gyrus of neurologically normal or clinically and pathologically diagnosed AD autopsy specimens were used (See Supplementary Table 2 for details). These were grown in the same conditions as epilepsy-derived pericytes, and both epilepsy and autopsy-derived pericytes have been previously validated to recapitulate a pericyte phenotype in vitro^31,68^. Pericyte cultures ubiquitously express canonical pericyte markers including PDGFRβ, CD13, CD146 and NG-2 and have a stable phenotype up to passage 10, and have been extensively characterised previously^56,63,69–71^. Furthermore, pericyte cultures generated this way have a consistent secretome, and pattern of response to diverse immune stimuli as we have described previously^56,69–71^. Cells grown here were derived from the middle temporal gyrus, however, we have cultured cells from other regions such as the hippocampus, and see similar phenotype and inflammatory response^72^.

### Primary human endothelial cell cultures

Primary human brain endothelia were grown as described previously^14^. In brief, microvessel fragments from a variety of surgical specimens (Supplementary Table 3) were plated onto Matrigel®-coated (Corning, NY, USA) flasks in Endothelial Cell Medium (ScienCell). Contaminating cell types were removed with a 1- to 2-week puromycin incubation (0.5–2 μg/mL; Sigma, MO, USA), before puromycin was removed. Endothelia were then subcultured with StemPro® Accutase®(Gibco), and seeded at 30,000 cells/cm^2^ into Matrigel®-coated plates for 1 week before harvest for qPCR experiments.

### RNA sequencing

RNA was harvested from neurologically normal or AD autopsy-derived human brain pericytes with two wells of a six-well plate harvested per condition as described previously^31^. RNA was extracted with the RNeasy® mini kit (Qiagen, Netherlands) as per manufacturer’s instructions from duplicate wells. At least 3 μg of RNA was desiccated in an RNAstable® tube (Biomatricia), and sent for Illumina sequencing (Novogene Ltd). Samples quality was verified with Nanodrop, Agarose Gel Electrophoresis and Agilent 2100. Any samples with a RIN < 8, low quantity or purity was discarded. After quality control, mRNA was enriched for each sample using oligo(dT) beads and Ribo-Zero kits. The mRNA is then fragmented, synthesised into cDNA and ligated with adaptors to prepare sequencing libraries. Qualified libraries are then pooled fed into Illumina sequencers (Novaseq 6000). Results were returned as pair-ended sequenced reads in a FASTQ file format. The raw data were filtered for adaptors, low quality reads and polyA sequences. Each sample achieved over 50 million, 150 bp paired-end reads after filtering and checked for quality with FastQC. Analysis of RNAseq data utilised the pipeline developed by Pertea et al, 2016 with HISAT2 (v2.1.0), StringTie (v1.3.4) and Ballgown (R version 3.5.1). In brief, paired-end reads from each sample was aligned to the reference human genome, *Homo Sapiens* GRCh38, using HISAT2, with all samples achieved alignment rate above 95%. The aligned reads are then assembled into genes and transcripts and quantified using StringTie. Finally, transcript abundance count tables are generated with the Ballgown R package. Differentially expressed genes were identified as having FPKM > 8 in one group, adjusted *p* value < 0.05, and log_2_ fold change >2. Gene lists were imputed into ENRICHR (https://amp.pharm.mssm.edu/Enrichr/) to provide gene ontology analysis, and STRING (https://string-db.org/) for protein network analysis. To identify if PDGF-BB altered pericyte/vSMC phenotype of cells, the top 200 most differentially expressed genes in pericytes vs venous SMC/arteriolar SMC/arterial SMC were collated from a vascular single-cell RNAseq database (http://betsholtzlab.org/VascularSingleCells/database.html). Genes were then classified as either pericyte or SMC, and expression changes at 24 h were ranked to provide Fig. 2i. Data are available from the Gene Expression Omnibus (GSE189712).

### Cytokine and drug treatments

Cells were treated with recombinant human PDGF-BB, IL-1β, TGF-β_1_, IFN-γ or their respective vehicles (see Table 1) as a 100-fold dilution of a 100× stock. For experiments involving sunitinib, crenolanib, CP-673451, wortmannin, U0126, TPCA-1 or vehicle (30% imethyl sulfoxide (DMSO) in Dulbecco’s Modified Eagle Medium (DMEM)), inhibitors were administered 2 h prior to cytokine treatments as a 100-fold dilution of a 100× stock. To stimulate apoptosis, pericytes were treated with okadaic acid (50 nM, 24 h) or vehicle (0.1 % DMSO) following a 24 h pre-treatment with either vehicle or PDGF-BB. Details for each experiment are given in appropriate figure legends. For western blot experiments in Fig. 4h–k, 100 ng/mL PDGF-BB was used, rather than 10 ng/mL, in order to improve the phosphorylated PDGFRβ signal.

**Table 1:** Specifications for cytokines and inhibitors used.

Immunogen	Activates	Conc. (range) (ng/mL)	Incubation (range) (h)	Supplier	Cat
IFN-γ	IFNGR	10	8 (0.25–8)	R&D, MN, USA	285-IF
IL-1β	IL-1R	10	24 (0.25–48)	PeproTech, NJ, USA	200-01B
PDGF-BB	PDGFRα/β	10 (0.01–100)	48 (0.25–96)	R&D, MN, USA	220-BB
TGF-β_1_	ALK1/5	10 (0.1–10)	8 (0.25–8)	PeproTech, NJ, USA	100-21

Compound	Inhibits	Conc. (range) (µM)	Incubation (range) (h)	Supplier	Cat
Sunitinib	PDGFRβ	0.1	>2	Cayman, MI, USA	19170
Crenolanib	PDGFRβ	0.1	>2	Selleckchem, MA, USA	CP-868596
CP-673451	PDGFRβ	0.1	>2	Sigma, MO, USA	PZ0012
Wortmannin	PI3K	0.1	>2	Cell Signalling, MA, USA	9903
U0126	MEK/ERK	10	>2	Sigma, MO, USA	W1628
TPCA-1	IκK	10	>2	Tocris, UK	2559

Most common incubations and concentrations are given, with ranges in brackets where appropriate.

### siRNA transfection

Synthetic siRNA targeted against the human *PDGFRB* gene (siPDGFRB; Santa Cruz, sc-29442), *RELA* gene (siRELA; Santa Cruz, sc-29410), *EGR1* gene (siEGR1; Sigma, NM_001964) or appropriate control siRNAs (Control siRNA-A; Santa Cruz, sc-37007/MISSION® siRNA Universal Negative Control #1; Sigma, SIC001) were incubated with Lipofectamine® RNAiMAX (Life Technologies) in OptiMEM (Gibco) for 20 min at room temperature. Lipofectamine:siRNA mixture was added to cells at a final concentration of 50 nM siRNA, as a five-fold dilution of a 5× stock. Cells were incubated for 96 h before treatment in order to achieve optimal knockdown (Supplementary Fig. 1, 5–6).

### Immunocytochemistry

Cells in 96-well plates were fixed in 4% paraformaldehyde (PFA) for 15 min, and when endosomal markers (EEA1, Rab5, Rab7, LAMP1) were stained, defatted with ice-cold methanol for a further 10 min at 4 °C. Cells were washed with PBS-Triton™ X-100 (0.1%; Sigma) (PBS-T). Primary antibodies, diluted appropriately in immunobuffer (PBS with 0.2% Triton™ X-100, 1% goat serum (Gibco), and 0.04% thimerosal (Sigma)), were added overnight at 4 °C (See Supplementary Table 4 for details). The following day, species-specific secondary antibodies and Hoechst nuclear counterstain (1 µM; Hoechst 33258, Sigma) in immunobuffer were added at room temperature for three hours. Wells were then washed again, and images were acquired on an ImageXpress Micro XLS™ automated microscope (Molecular Devices).

### Image analysis for cell staining

Image analysis was performed on MetaXpress™ (v5.3.04) software.

#### Cell counts

Nuclei were thresholded based on Hoechst staining and size (5–25 µm diameter) and total cell numbers per field of view were presented.

#### Percentage positive

Cells positive for MCP-1, IL-6, EdU, Ki67 and NucGreen® Dead were defined as staining associated with a nucleus above a thresholded positive level, and expressed as a percentage of the total cell number.

#### Stain intensity quantification

To quantify the expression of PDGFRβ, EGR-1, SMAD2/3, STAT1, STAT3 and cJun, a low threshold was set to exclude background staining, then the integrated intensity of the stain was measured. Values were expressed normalised to cell counts to account for changes in the number of cells.

#### Scratch assay migration

The ability of cells to migrate into the gap was measured using an inclusive threshold. The gap area was quantified as the percentage thresholded area out of the total field of view.

#### Puncta formation assay

The formation of PDGFRβ puncta was measured based on the granularity of PDGFRβ staining, using a previously validated assay^73^. Settings were optimised to detect small puncta and expressed as puncta per cell.

#### Nuclear translocation assay

The translocation of NF-κB to the nucleus was measured using a journal that detects the correlation between a cellular compartment stain and a stain of interest. Correlation between the compartment stain (Hoechst) and NF-κB was set above 0.7 for each cell. Data were expressed as a percentage of translocating cells.

#### Puncta colocalisation assay

Colocalization of PDGFRβ-positive puncta and endosomes was performed using the custom module editor within MetaXpress, and optimised to detect small bright features overlapping one another.

### Scratch wound-healing assay

A confluent monolayer of pericytes was scratched with a P200 pipette tip. Cells were washed two times in warm PBS. Cells were then given fresh media with or without mitotic inhibitors cytosine β-d-arabinofuranoside (AraC, 1 µM; Sigma), 5-fluoro-2’-deoxyuridine (FdUR, 1 µM; Sigma), uridine (Urd, 1 µM; Sigma). Pericytes were treated with vehicle or sunitinib, wortmannin, U0126 or TPCA-1 for 2–4 h (see Table 1 for details), then treated with vehicle or PDGF-BB and left to migrate into the scratch for a further 48 h. Cells were then fixed in 4% PFA for 15–20 min and washed in PBS-T. Cells were stained with Coomassie Brilliant Blue R250 (0.25% w/v in 10% glacial acetic acid, 45% methanol; Gibco, CA, USA) for 30 min. The stain was then removed and cells were allowed to air dry. Wells were imaged at 4× magnification using brightfield imaging on the ImageXpress Micro XLS microscope.

### 5-ethynyl-2’-deoxyuridine (EdU) proliferation assay

Proliferation was measured by 5-ethynyl-2’-deoxyuridine (100 nM; Click-iT® EdU AlexaFluor® 647 Imaging Kit; Molecular Probes, CA, USA) incorporation for 48 h at 37 °C, 5% CO_2_. Fixed cells that had been immunostained, as described above, were then washed in 3% BSA in PBS-T twice. EdU staining was visualised using the Click-iT® AlexaFluor™ 647 kit as per the manufacturer’s instructions. Nuclei positive for EdU were scored using MetaXpress™ version 5.3.04 (Molecular Devices) analysis software.

### AlamarBlue viability assay

To determine cell viability, AlamarBlue® (1:10 final dilution in culture medium; Life Technologies, CA, USA) was added to cells in a 96-well plate for 2 h at 37 °C, 5% CO_2_. Fluorescence (Ex 544/ Em 590) was read on a CLARIOstar monochromator microplate reader (BMG Labtech, Germany). Background fluorescence of wells with media but no cells was subtracted from all values. Fluorescence intensity values were then normalised to vehicle control.

### ReadyProbes live/dead viability assay

Cell death was assessed using the ReadyProbes™ Cell Viability Imaging Kit, Blue/Green. Briefly, media containing NucBlue® Live and NucGreen® Dead (two drops per mL) was added to wells, and incubated for 30 min at 37 °C before imaging on an ImageXpress® Micro XLS (version 5.3.0.1, Molecular Devices, CA, USA) as described above. Dead cells were scored based on NucGreen® Dead nuclear staining, and apoptosis was expressed as the percentage of NucGreen®-positive cells. Conversely, viability was expressed as total NucBlue®-positive cells minus dead cells.

### Western blot

Cells from a single well of a six-well plate were harvested into Eppendorf tubes by scraping. Cells were centrifuged, and pellet was resuspended in lysis buffer (25 mM Tris-HCl pH 7.5, 150 mM NaCl, 50 mM NaF, 0.5 mM EDTA pH 8, 0.5 % Triton-X-100™, 5 mM β-glycerophosphate, with fresh 1 mM DTT, 1 mM PMSF, 1 mM Na_3_VO_4_). Before being run on a gel, lysates were diluted 1:1 in 2× modified Laemmli buffer (125 mM Tris-HCl, pH 6.8, 5% glycerol, 4% sodium dodecyl sulphate (SDS), 0.2% bromophenol blue (w/v)). Lysates were separated on a 4–12% pre-cast gel by electrophoresis, and transferred to a polyvinylidene fluoride membrane (Millipore (Billerica, MA, USA) IPFL00010 Immobilon-FL 0.45 mm). Blotting was performed as previously described by blocking the membrane in Odyssey blocking buffer (Li-COR, NE, USA) for 1 hour at room temperature^28^. Primary antibodies, diluted appropriately in 1:1 Odyssey: tris-buffered saline with 1% tween (TBST) were added overnight at 4 °C (See Supplementary Table 4 for details). Membranes were then incubated with fluorescently conjugated secondary antibodies diluted in Odyssey:TBST with 0.01% SDS for 2 hours at room temperature in the dark. Images were captured using a Li-COR Odyssey FC® imaging system, and band intensity was quantified using Image Studio™ Lite (Ver 5.0). Images were converted to greyscale and inverted on Adobe Photoshop. Full membranes from which representative blots were taken are shown in Supplementary Fig. 8.

### Flow cytometry

Cells grown in a 12-well plate were dissociated at endpoint using StemPro® Accutase® (5 min, 37 °C; Gibco), and the dissociation was quenched by the addition of complete media. For cell-surface labelling of PDGFRβ, cells were centrifuged (160 × *g*, 5 min) and resuspended in cold flow cytometry buffer (1% FBS in PBS) as a wash. Cells were centrifuged (160 × *g*, 5 min), and supernatant discarded before 7-aminoactinomycin D (5 µL per test, 7-AAD; 51-68981E, BD Biosciences) and mouse anti-human CD140b-PE (10 µL per test; 558821, BD Biosciences) or mouse IgG2a-κ-PE isotype control (10 µL per test; 555574, BD Biosciences) were added to suspended cells on ice for 15 min^68^. Cold flow cytometry buffer was added, then samples were spun (160 × *g*, 5 min), the supernatant discarded and fluorescence measured using an Accuri C6 flow cytometer (BD Biosciences). At least 10,000 cellular events were gated based on forward and side scatter, and 7-AAD exclusion. Gating is shown in Supplementary Fig. 9.

To measure total PDGFRβ expression by flow cytometry, cells were harvested using Accutase® as above, then fixed by addition of an equal volume of 8% PFA. Cells were incubated for 15–20 min, then washed twice in PBS-T to permeabilise. Cells were spun (160 × *g*, 5 min) and the supernatant discarded, then anti-CD140b (1:500 in goat immunobuffer; 7460–3104, BioRad) added overnight at 4 °C with agitation. The following day, cells were washed with PBS-T, then centrifuged (160 × *g*, 5 min) and secondary antibody (1:500; goat anti-mouse AlexaFluor®-488) and Molecular Probes™ NucRed® (2 drops per mL) added for three hours at room temperature. Cells were washed, centrifuged (160 × *g*, 5 min) and the supernatant discarded, then fluorescence was measured using an Accuri C6 flow cytometer (BD Biosciences) with at least 10,000 cellular events gated, based on forward and side scatters, and NucRed® inclusion. Analysis of flow cytometry data was performed using FlowJo software (v 7.6.5). Mean fluorescence intensity of CD140b cell-surface expression was determined from three independent cases. Gating is shown in Supplementary Fig. 9. A small volume of fixed cell suspension was mounted on a slide and imaged with a ×40 objective on a Nikon Eclipse N*i* microscope to generate the panel in Fig. 3k (NA 0.95; Japan).

### Cytometric bead array

Conditioned media samples were centrifuged (160 × *g*, 5 min), the supernatant collected and were stored at −20 °C until analysis. The concentration of cytokines was measured using cytometric bead array (CBA; BD Biosciences, CA, USA) as per manufacturer’s instructions (See Supplementary Table 5 for CBA kit details). CBA samples were run on an Accuri C6 flow cytometer (BD Biosciences, CA, USA). Data were analysed using FCAP-array software (version 3.1; BD Biosciences, CA, USA) to convert fluorescent intensity values to concentrations using an eleven-point standard curve (0–10,000 pg/mL) and normalised to cell number.

### Quantitative polymerase chain reaction

Total RNA was extracted from cells grown in a 12-well plate using the Ambion RNA Micro-scale extraction kit (Ambion, CA, USA) following the manufacturer’s instructions. For complementary DNA (cDNA) synthesis, 500 ng total cDNA was made per sample. RNA was added to each reaction mix accordingly and diluted to give the same volume, before deoxyribonuclease I (DNase I) treatment (RQ1 RNase-free DNase kit; Promega, WI. USA) with 1 µg DNase added per 1 µg RNA. The Superscript III First-Strand Synthesis kit (Life Technologies, CA, USA) was used for cDNA synthesis. Quantitative real-time PCR was performed using Platinum SYBR Green qPCR SuperMix-UDG with Rox (Life Technologies, CA, USA) on a 7900HT Fast Real-Time PCR system (Applied Biosystems, Life Technologies, CA, USA) (see Supplementary Table 6 for primer details). Relative gene expression was graphed as fold enrichment using the 2^−ΔΔCt^ method.

### NanoString

RNA was extracted from fresh-frozen human brain tissue taken from the middle frontal gyrus of post-mortem control (*N* = 8) and AD (*N* = 8) cases (Supplementary Table 7). Care was taken to dissect only grey matter from the tissue samples (<30 mg), which were then immediately homogenised in 1 mL TRIzol reagent with 2 mm stainless steel beads (Qiagen) using the Tissuelyser II tissue homogeniser (Qiagen) for 4 min at 25 Hz. Samples were centrifuged at 12,000 × *g* for 2 min at 4 °C. The supernatant was collected and 200 μL of chloroform added, shaken vigorously for 15 s and incubated for 2–3 min at RT and then centrifuged at 12,000 × g for 15 min at 4 °C. The upper aqueous phase was collected and mixed with an equal volume of 70% ethanol. Subsequent steps were performed using the RNeasy kit (Qiagen) following the manufacturer’s instructions. DNase I treatment was performed using components from the RNAqueous-Micro kit (Ambion) following the manufacturer’s instructions. RNA purity and concentrations were determined using Qubit and Bioanalyzer 2100 (Agilent Technologies). Only samples with RIN > 5 were used for Nanostring. Samples were shipped on dry ice to the Otago Genomics Facility (Otago, NZ) for further QC and processing on the Nanostring N-Counter using a custom CodeSet that included five reference housekeeping genes (*ACTB*, *PGK1*, *POL1B*, *RPLP0* and *RPL30*) which were used for normalisation. All data passed QC, with no imaging, binding, positive control or CodeSet content normalisation flags. Background-corrected counts (mean +1 SD) normalised to the geometric mean of both the positive controls (between lane hyb effects) and all nominated reference, housekeeping genes (RNA input effects) for all samples were used for graphs.

### Publicly available RNAseq data

Patient details and FPKM values for *PDGFB* and *PDGFRB* were extracted from the Mayo Clinic late-onset AD temporal cortex RNAseq data set (10.7303/syn3163039)^33^. Patients denoted as ‘Control’ (*n* = 78) and ‘Alzheimer’s disease’ (*n* = 82) were included for analysis in Fig. 1f.

### Statistics and reproducibility

Data from tissue staining, ISH and AD patient RNAseq are presented as box and whiskers, with the box representing the interquartile range, whiskers 10–90 percentiles, outliers as dots and a bar at the median. All cell-based experiments were performed in at least three independent pericyte/endothelial lines derived from biopsy/autopsy specimens. Data from in vitro experiments are expressed as mean ± standard error of mean from at least three independent experiments. Data visualisation and statistical hypothesis testing were performed using GraphPad Prism® Version 7.00. Two-way analysis of variance (ANOVA) was used when comparing across levels of two factors with Tukey’s post-hoc adjustment for multiple comparisons, whereas one-way ANOVA with Dunnett’s multiple comparison adjustment was used when comparing between levels of one factor. A Student’s *t* test was performed to compare the differences between two groups. Statistical tests for sets of experiments are denoted in the figure legends. For pathological studies, absolute *p* values are given. Statistical significance is denoted as follows: *p < 0.05, **p < 0.01, ***p < 0.001 vs vehicle control, ^#^*p* < 0.05, ^##^*p* < 0.01, ^###^*p* < 0.001 vs PDGF-BB-treated.

### Reporting summary

Further information on research design is available in the Nature Research Reporting Summary linked to this article.

## Supplementary information

Supplementary Material

Description of Additional Supplementary Files

Supplementary Data

Reporting Summary

## Data Availability

Source data can be found in the Supplementary Data file. RNAseq data are available from the Gene Expression Omnibus (GSE189712). The data presented in this study are included in the manuscript and supplementary material. Additional data that are not included can be made available upon reasonable request to the corresponding author.

## References

[1] Zlokovic BV (2005). Neurovascular mechanisms of Alzheimer’s neurodegeneration. Trends Neurosci..

[2] Yang, A. C. et al. A human brain vascular atlas reveals diverse cell mediators of Alzheimer’s disease risk. *bioRxiv*10.1101/2021.04.26.441262 (2021).

[3] Halliday MR (2016). Accelerated pericyte degeneration and blood–brain barrier breakdown in apolipoprotein E4 carriers with Alzheimer’s disease. J. Cereb. Blood Flow. Metab..

[4] Zhang X (2019). High-resolution mapping of brain vasculature and its impairment in the hippocampus of Alzheimer’s disease mice. Natl. Sci. Rev..

[5] Iturria-Medina Y (2016). Early role of vascular dysregulation on late-onset Alzheimer’s disease based on multifactorial data-driven analysis. Nat. Commun..

[6] Nortley, R. et al. Amyloid-beta oligomers constrict human capillaries in Alzheimer’s disease via signaling to pericytes. *Science* **365**, eaav9518 (2019). 10.1126/science.aav9518. PMC6658218 31221773

[7] Nation, D. A. et al. Blood–brain barrier breakdown is an early biomarker of human cognitive dysfunction. *Nat. Med.*10.1038/s41591-018-0297-y (2019). 10.1038/s41591-018-0297-y PMC6367058 30643288

[8] Montagne, A. et al. APOE4 leads to blood–brain barrier dysfunction predicting cognitive decline. *Nature*10.1038/s41586-020-2247-3 (2020). 10.1038/s41586-020-2247-3 PMC7250000 32376954

[9] Sagare AP (2013). Pericyte loss influences Alzheimer-like neurodegeneration in mice. Nat. Commun..

[10] Daneman R, Zhou L, Kebede AA, Barres BA (2010). Pericytes are required for blood–brain barrier integrity during embryogenesis. Nature.

[11] Bourassa, P., Tremblay, C., Schneider, J. A., Bennett, D. A. & Calon, F. Brain mural cell loss in the parietal cortex in Alzheimer’s disease correlates with cognitive decline and TDP‐43 pathology. *Neuropathol. Appl. Neurobiol.*10.1111/nan.12599 (2020). 10.1111/nan.12599 PMC7739958 31970820

[12] Montagne A (2015). Blood-brain barrier breakdown in the aging human hippocampus. Neuron.

[13] Lindahl P, Johansson BR, Levéen P, Betsholtz C (1997). Pericyte loss and microaneurysm formation in PDGF-B-deficient mice. Science.

[14] Smyth LCD (2018). Unique and shared inflammatory profiles of human brain endothelia and pericytes. J. Neuroinflamm..

[15] Vanlandewijck M (2018). A molecular atlas of cell types and zonation in the brain vasculature. Nature.

[16] Armulik A (2010). Pericytes regulate the blood–brain barrier. Nature.

[17] Paul G (2012). The adult human brain harbors multipotent perivascular mesenchymal stem cells. PLoS ONE.

[18] Lindblom P (2003). Endothelial PDGF-B retention is required for proper investment of pericytes in the microvessel wall. Genes Dev..

[19] Nakamura K (2019). Perlecan regulates pericyte dynamics in the maintenance and repair of the blood-brain barrier. J. Cell Biol..

[20] Bell RD (2010). Pericytes control key neurovascular functions and neuronal phenotype in the adult brain and during brain aging. Neuron.

[21] Nikolakopoulou AM, Zhao Z, Montagne A, Zlokovic BV (2017). Regional early and progressive loss of brain pericytes but not vascular smooth muscle cells in adult mice with disrupted platelet-derived growth factor receptor-β signaling. PLoS ONE.

[22] Vazquez-Liébanas, E. V. et al. Endothelium-derived PDGF-B is essential for mural cell maintenance and endothelial cell quiescence in the adult brain. *Cold Spring Harb. Brain Barriers* **185** (2021).

[23] Arango-Lievano M (2018). Topographic reorganization of cerebrovascular mural cells under seizure conditions. Cell Rep..

[24] Geraldes P (2009). Activation of PKC-δ and SHP-1 by hyperglycemia causes vascular cell apoptosis and diabetic retinopathy. Nat. Med..

[25] Chintalgattu V (2013). Coronary microvascular pericytes are the cellular target of sunitinib malate-induced cardiotoxicity. Sci. Transl. Med..

[26] Ma Q (2018). Blood-brain barrier-associated pericytes internalize and clear aggregated amyloid-β42 by LRP1-dependent apolipoprotein E isoform-specific mechanism. Mol. Neurodegener..

[27] Sagare AP, Sweeney MD, Makshanoff J, Zlokovic BV (2015). Shedding of soluble platelet-derived growth factor receptor-β from human brain pericytes. Neurosci. Lett..

[28] Jansson D (2016). Interferon-γ blocks signalling through PDGFRβ in human brain pericytes. J. Neuroinflamm..

[29] Gate, D. et al. Clonally expanded CD8 T cells patrol the cerebrospinal fluid in Alzheimer’s disease. *Nature*10.1038/s41586-019-1895-7 (2020). 10.1038/s41586-019-1895-7 PMC7445078 31915375

[30] Stubbing, L. A. et al. Synthesis of peptide homo‐ and heterodimers as potential mimics of platelet‐derived growth factor BB. *Peptide Sci.*10.1002/pep2.24150 (2020).

[31] Smyth, L. C. D. et al. Markers for human brain pericytes and smooth muscle cells. *J. Chem. Neuroanat.* (2018) 10.1016/j.jchemneu.2018.06.001 (2018). 10.1016/j.jchemneu.2018.06.001 29885791

[32] Highet, B. et al. fISHing with immunohistochemistry for housekeeping gene changes in Alzheimer’s disease using an automated quantitative analysis workflow. *J. Neurochem.* **157**, 1270–1283 (2021). 10.1111/jnc.15283 33368239

[33] Allen M (2016). Human whole genome genotype and transcriptome data for Alzheimer’s and other neurodegenerative diseases. Sci. Data.

[34] Allen M (2016). Gene expression, methylation and neuropathology correlations at progressive supranuclear palsy risk loci. Acta Neuropathol..

[35] Porsch H, Mehić M, Olofsson B, Heldin P, Heldin C-H (2014). Platelet-derived growth factor β-receptor, transforming growth factor β type I receptor, and CD44 protein modulate each other’s signaling and stability. J. Biol. Chem..

[36] He L (2016). Analysis of the brain mural cell transcriptome. Sci. Rep..

[37] Yamamoto H, Crow M, Cheng L, Lakatta E, Kinsella J (1996). PDGF receptor-to-nucleus signaling of p91 (STAT1α) transcription factor in rat smooth muscle cells. Exp. Cell Res..

[38] Jastrzębski K (2017). Multiple routes of endocytic internalization of PDGFRβ contribute to PDGF-induced STAT3 signaling. J. Cell Sci..

[39] Yu Y (2003). PDGF stimulates pulmonary vascular smooth muscle cell proliferation by upregulating TRPC6 expression. Am. J. Physiol. Cell Physiol..

[40] Lennartsson J (2013). The Fer tyrosine kinase is important for platelet-derived growth factor-BB-induced signal transducer and activator of transcription 3 (STAT3) protein phosphorylation, colony formation in soft agar, and tumor growth in vivo. J. Biol. Chem..

[41] Shen J (2012). PDGFR-β as a positive regulator of tissue repair in a mouse model of focal cerebral ischemia. J. Cereb. Blood Flow. Metab..

[42] Hellström M, Kalén M, Lindahl P, Abramsson A, Betsholtz C (1999). Role of PDGF-B and PDGFR- β in recruitment of vascular smooth muscle cells and pericytes during embryonic blood vessel formation in the mouse. Development.

[43] Vazquez-Liebanas, E. et al. Adult-induced genetic ablation distinguishes PDGFB roles in blood-brain barrier maintenance and development. *J. Cereb. Blood Flow Metab.*10.1177/0271678X211056395 (2021). 10.1177/0271678X211056395 PMC8795218 34689641

[44] Sweeney MD, Ayyadurai S, Zlokovic BV (2016). Pericytes of the neurovascular unit: key functions and signaling pathways. Nat. Neurosci..

[45] Zlokovic BV (2011). Neurovascular pathways to neurodegeneration in Alzheimer’s disease and other disorders. Nat. Rev. Neurosci..

[46] Jew B (2020). Accurate estimation of cell composition in bulk expression through robust integration of single-cell information. Nat. Commun..

[47] Yang, A. C. et al. Physiological blood–brain transport is impaired with age by a shift in transcytosis. *Nature*10.1038/s41586-020-2453-z (2020). 10.1038/s41586-020-2453-z PMC8331074 32612231

[48] Pfau, S. J. et al. Vascular and perivascular cell profiling reveals the molecular and cellular bases of blood-brain barrier heterogeneity. *bioRxiv*10.1101/2021.04.26.441465 (2021).

[49] Roth, M. et al. Regulator of G-protein signaling 5 regulates the shift from perivascular to parenchymal pericytes in the chronic phase after stroke. *FASEB J.*10.1096/fj.201900153R (2019). 10.1096/fj.201900153R PMC6662981 31039042

[50] Shen, J. et al. PDGFR-β restores blood-brain barrier functions in a mouse model of focal cerebral ischemia. *J. Cereb. Blood Flow Metabo.*10.1177/0271678X18769515 (2018). 10.1177/0271678X18769515 PMC6681529 29629621

[51] Schmahl J, Raymond CS, Soriano P (2007). PDGF signaling specificity is mediated through multiple immediate early genes. Nat. Genet..

[52] Wu J, Bohanan CS, Neumann JC, Lingrel JB (2008). KLF2 transcription factor modulates blood vessel maturation through smooth muscle cell migration. J. Biol. Chem..

[53] Deaton RA, Gan Q, Owens GK (2009). Sp1-dependent activation of KLF4 is required for PDGF-BB-induced phenotypic modulation of smooth muscle. Am. J. Physiol..

[54] Olson LE, Soriano P (2011). PDGFRβ signaling regulates mural cell plasticity and inhibits fat development. Dev. Cell.

[55] Romashkova JA, Makarov SS (1999). NF-κB is a target of AKT in anti-apoptotic PDGF signalling. Nature.

[56] Rustenhoven J (2016). TGF-beta1 regulates human brain pericyte inflammatory processes involved in neurovasculature function. J. Neuroinflamm..

[57] Rustenhoven J, Jansson D, Smyth LC, Dragunow M (2017). Brain pericytes as mediators of neuroinflammation. Trends Pharmacol. Sci..

[58] Kang, T.-Y. et al. Pericytes enable effective angiogenesis in the presence of proinflammatory signals. *Proc. Natl. Acad. Sci.*10.1073/pnas.1913373116 (2019). 10.1073/pnas.1913373116 PMC6876202 31685607

[59] Dohgu S (2005). Brain pericytes contribute to the induction and up-regulation of blood–brain barrier functions through transforming growth factor-β production. Brain Res..

[60] Chen J (2017). CD146 coordinates brain endothelial cell-pericyte communication for blood-brain barrier development. Proc. Natl. Acad. Sci. USA.

[61] Duan L (2018). PDGFRβ cells rapidly relay inflammatory signal from the circulatory system to neurons via chemokine CCL2. Neuron.

[62] Klement W (2019). A pericyte‐glia scarring develops at the leaky capillaries in the hippocampus during seizure activity. Epilepsia.

[63] Jansson D (2014). A role for human brain pericytes in neuroinflammation. J. Neuroinflamm..

[64] Waldvogel HJ, Curtis MA, Baer K, Rees MI, Faull RLM (2007). Immunohistochemical staining of post-mortem adult human brain sections. Nat. Protoc..

[65] Singh-Bains MK, Mehrabi NF, Tan AYS, Faull RLM, Dragunow M (2021). Preparation, construction and high-throughput automated analysis of human brain tissue microarrays for neurodegenerative disease drug development. Nat. Protoc..

[66] Narayan PJ (2015). Assessing fibrinogen extravasation into Alzheimer’s disease brain using high-content screening of brain tissue microarrays. J. Neurosci. Methods.

[67] Gibbons HM (2007). Cellular composition of human glial cultures from adult biopsy brain tissue. J. Neurosci. Methods.

[68] Rustenhoven J (2018). Modelling physiological and pathological conditions to study pericyte biology in brain function and dysfunction. BMC Neurosci..

[69] Park TI-H (2016). Cultured pericytes from human brain show phenotypic and functional differences associated with differential CD90 expression. Sci. Rep..

[70] Rustenhoven J (2015). An anti-inflammatory role for C/EBPδ in human brain pericytes. Sci. Rep..

[71] Jansson D (2021). Cardiac glycosides target barrier inflammation of the vasculature, meninges and choroid plexus. Commun. Biol..

[72] Park TI-H (2012). Adult human brain neural progenitor cells (NPCs) and fibroblast-like cells have similar properties in vitro but Only NPCs differentiate into neurons. PLoS ONE.

[73] Grimsey NL, Narayan PJ, Dragunow M, Glass M (2008). A novel high-throughput assay for the quantitative assessment of receptor trafficking. Clin. Exp. Pharmacol. Physiol..

