# Supplementary material for: Characterisation of PDGF-BB:PDGFRβ signalling pathways in human brain pericytes: evidence of disruption in Alzheimer’s disease
Source: Commun Biol. 2022 Mar 17;5:235. doi: 10.1038/s42003-022-03180-8 (PMC8931009; doi:10.1038/s42003-022-03180-8)
Supplement: Supplementary file 1 — Supplementary Material [file 42003_2022_3180_MOESM1_ESM.pdf]

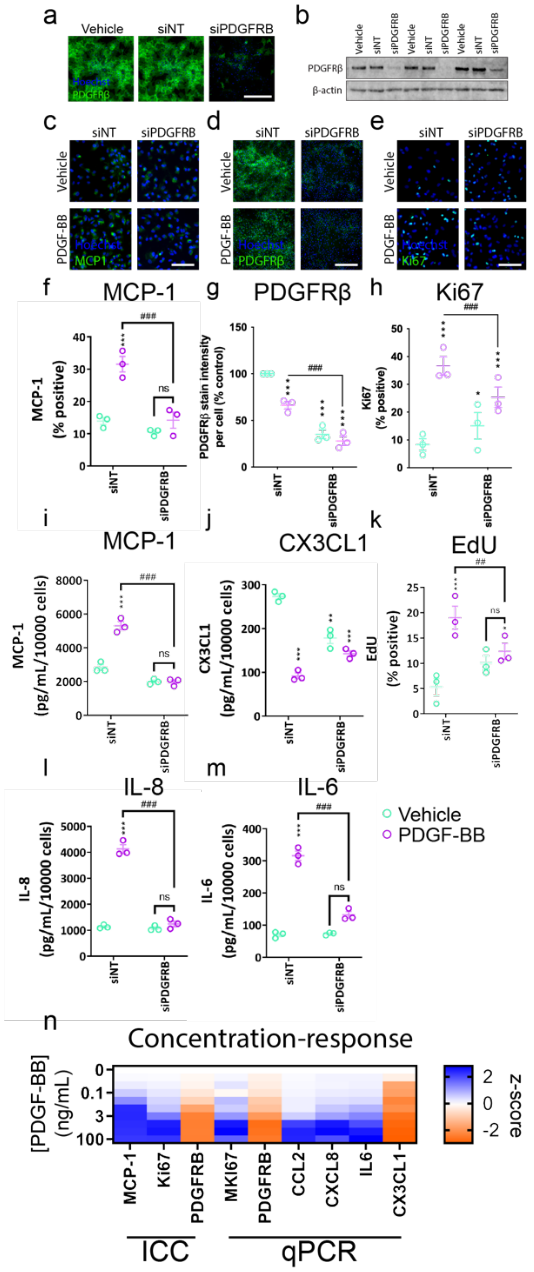

Supplementary Figure 1: PDGF-BB acts through PDGFRβ in pericytes. Pericytes were treated with Lipofectamine (vehicle; 0.03%), non-targeted siRNA, or siRNA against PDGFRβ (50 nM, 96 h). Cells were then fixed for immunostaining or lysed for western blot. a) Immunostaining and b) immunoblot of PDGFRβ knockdown in pericytes. Scale bar = 250 µm. Pericytes were treated with non-targeted siRNA or siRNA against PDGFRβ (50 nM, 96 h), then treated with PDGF-BB for 2, 24, or 48 hours and cells were fixed and immunostained or conditioned media taken for cytometric bead array. EdU was added 24 hours before endpoint. Representative images and quantification of c, f) MCP-1, d, g) PDGFRβ, e, h) Ki67, and k) EdU staining in pericytes treated with PDGF-BB with or without siRNA against PDGFRβ. Scale bar = 100 µm. Quantification of i) MCP-1, j) CX3CL1, l) IL-8, and m) IL-6 secretion in pericytes treated with PDGF-BB with or without siRNA against PDGFRβ. Pericytes were treated with serially diluted PDGF-BB (range 0.01-100 ng/mL), then fixed and immunostained or RNA extracted for qPCR. n) Heatmap of z-scores of PDGF-BB responses across the concentration range. n = 3, two way ANOVA. * - $p < 0.05$, ** - $p < 0.01$, *** - $p < 0.001$ vs vehicle control, # - $p < 0.05$, ## - $p < 0.01$, ### - $p < 0.001$ vs PDGF-BB-treated.

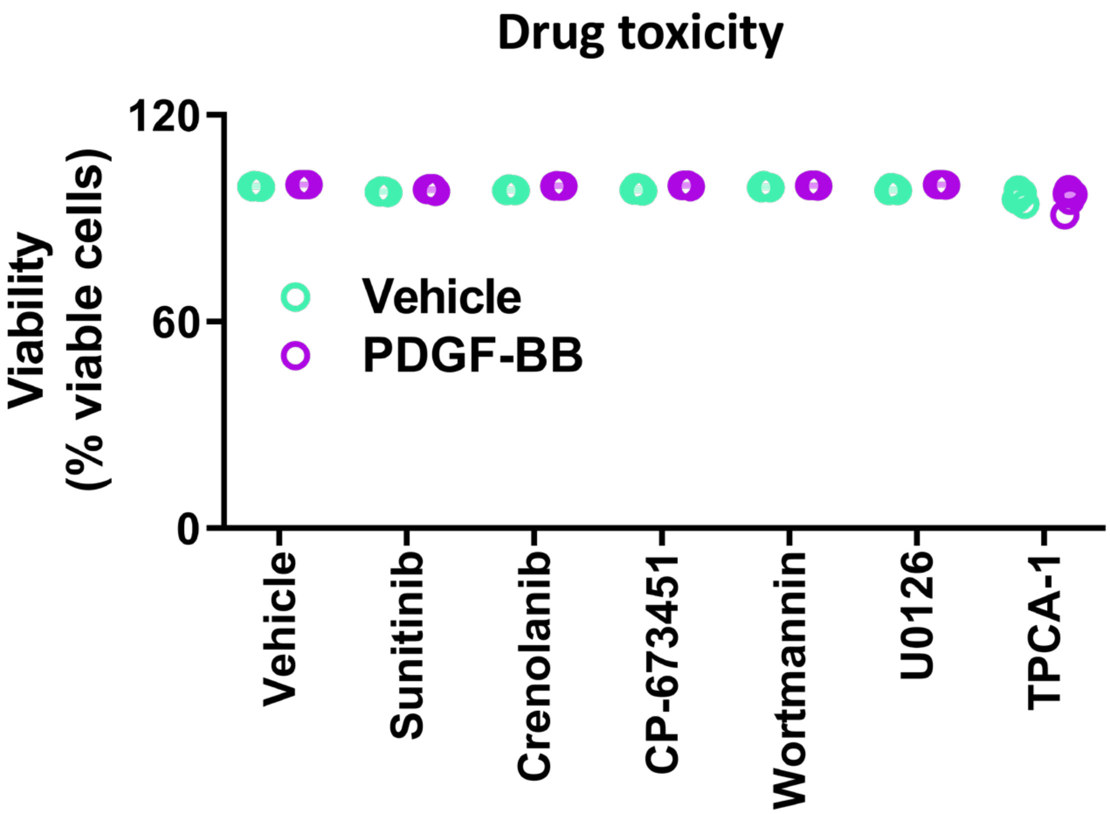

Supplementary Figure 2: Toxicity of PDGFRβ, PI3K, ERK, and NF-κB inhibitors to pericytes. Pericytes were treated with vehicle (0.3% DMSO) or inhibitors of PDGFRβ (sunitinib, crenolanib, CP-673451; 100 nM), PI3K (wortmannin; 100 nM), MEK/ERK (U0126; 10 µM), or IκK/NF-κB (TPCA-1; 10 µM) for 30 minutes, then vehicle or PDGF-BB (10 ng/mL) for 48 hours. Viability was quantified by the ReadyProbes[TM] viability imaging kit. n = 3.

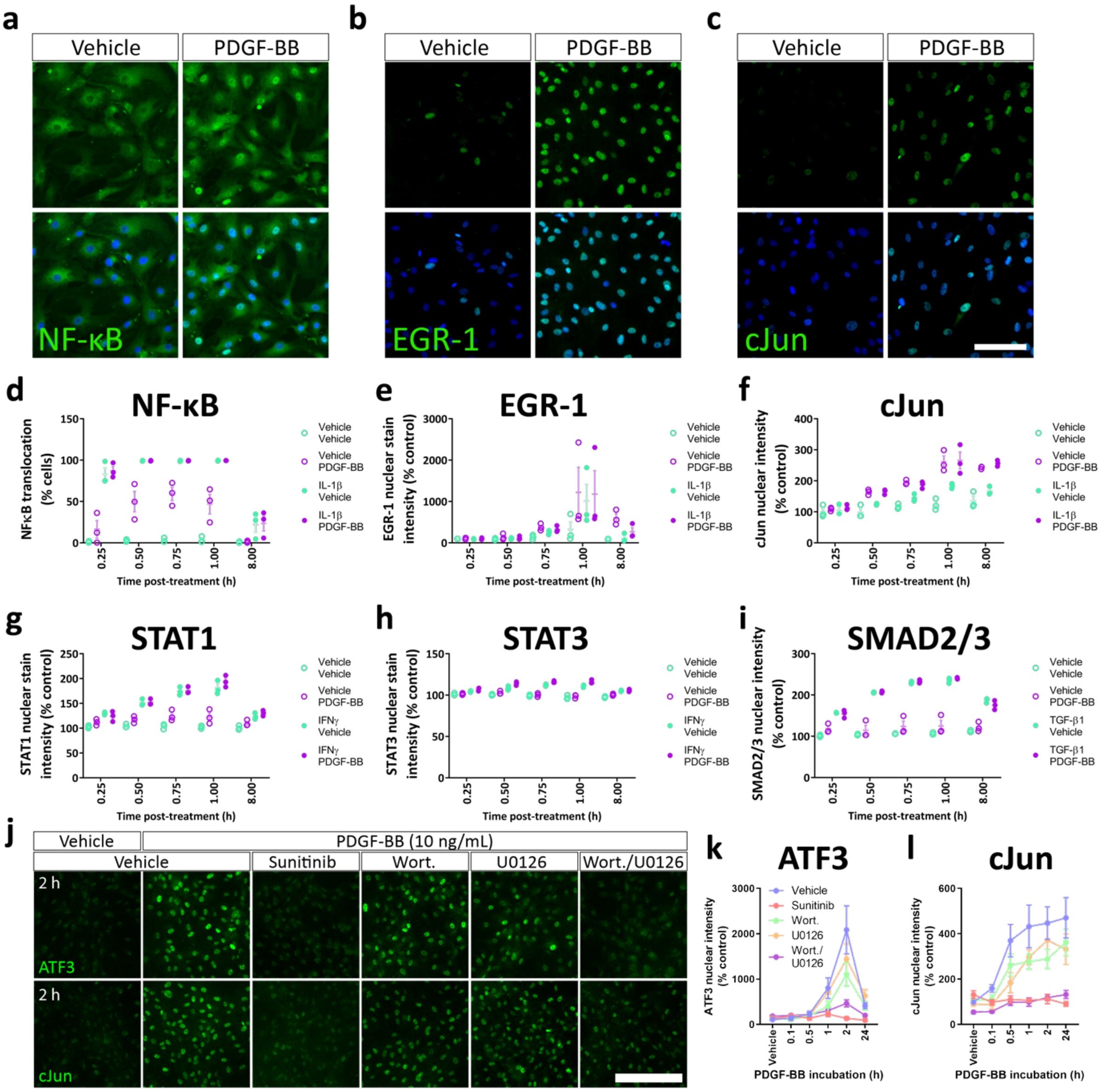

Supplementary Figure 3: PDGF-BB activates NF-κB, EGR-1, cJun, and ATF3 but not STAT1, STAT3, or SMAD2/3. Pericytes were treated with IL-1β (10 ng/mL; NF-κB/EGR-1/cJun), IFNγ (10 ng/mL; STAT1/STAT3), and TGF-β_1 (10 ng/mL; SMAD2/3) with or without PDGF-BB (10 ng/mL) for 0.25-8 h. Cells were then fixed and immunostained. Representative images and quantification of a, d) NF-κB, b, e) EGR-1, c, f) cJun, g) STAT1, h) STAT3, and i) SMAD2/3 immunostaining in pericytes treated with PDGF-BB, with or without the positive control cytokine. Scale bar = 50 µm. Pericytes were pre-treated with PDGFRβ inhibitor sunitinib (100 nM), PI3K inhibitor wortmannin (100 nM), MEK/ERK inhibitor U0126 (10 µM), both wortmannin and U0126, or vehicle (0.3% DMSO) for 30 minutes, then treated with vehicle or PDGF-BB (10 ng/mL) for up to 24 hours, then fixed and immunostained. j) Representative images and quantification of k) ATF3 and l) cJun immunostaining in pericytes treated with PDGF-BB, with or without pathway inhibitors. Scale bar = 200 µm. n = 3. * - p < 0.05, ** - p < 0.01, *** - p < 0.001 vs vehicle control, # - p < 0.05, ## - p < 0.01, ### - p < 0.001 vs PDGF-BB-treated.

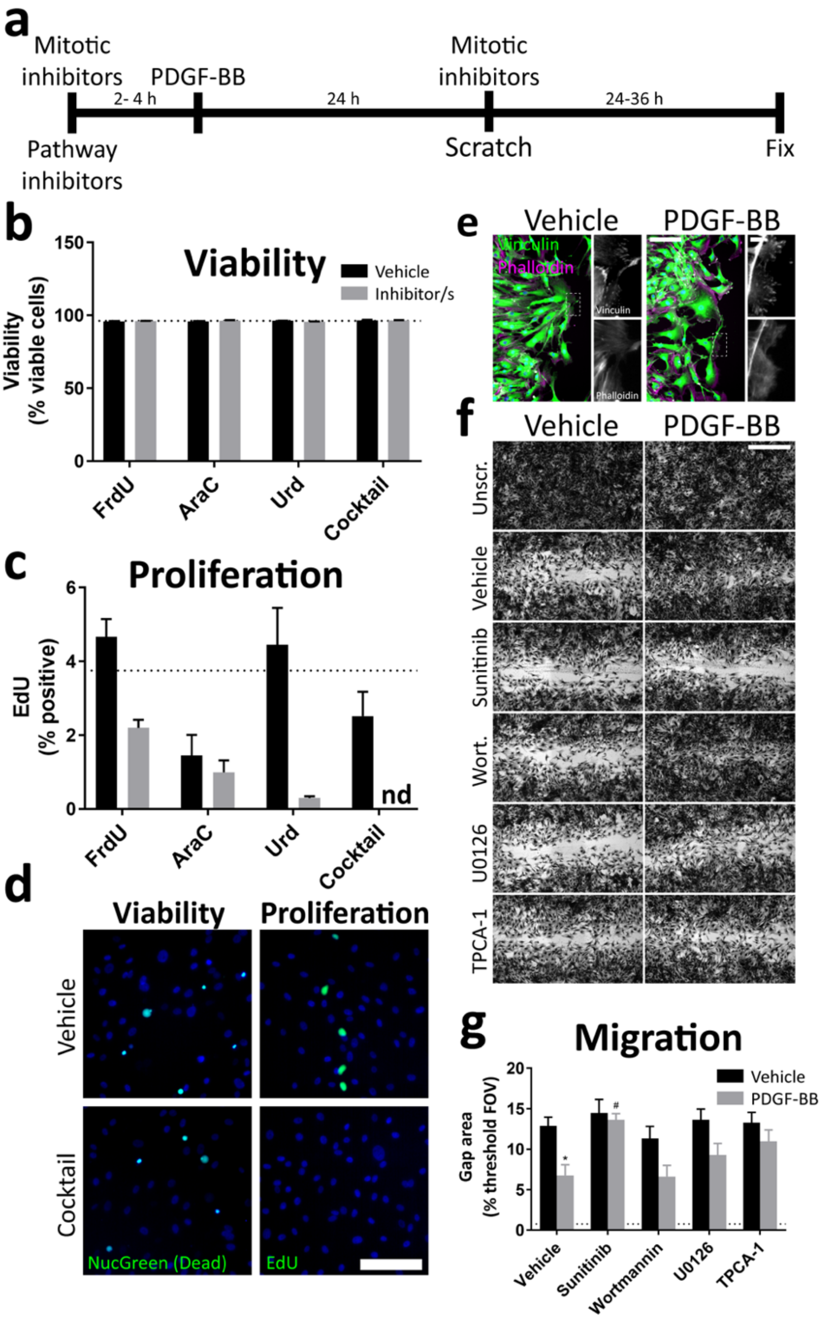

Supplementary Figure 4: PDGF-BB promotes pericyte migration independently of proliferation. Brain pericytes were treated with mitotic inhibitors FrdU, AraC, Urd, or a cocktail containing all of them. Cells were then treated with EdU for 24 h, and viability and proliferation measured by the ReadyProbes^{TM} viability imaging kit and EdU assay, respectively. a) Experimental design for migration experiments. Effect of mitotic inhibitors on b) viability and c) proliferation of pericytes, and d) representative images. Scale bar = 100 µm. Pericytes were treated with the mitotic inhibitor cocktail, with or without sunitinib (100 nM), wortmannin (100 nM), or U0126 (10 µM), then incubated with PDGF-BB (10 ng/mL) for a further 24 h. Cell monolayers were then scratched, and allowed 24-36 hours to migrate into the gap, and fixed at 2 hours for immunostaining or once one group had sealed the gap for migration experiments. e) Representative actin and vinculin immunostaining in leading pericytes treated with PDGF-BB or vehicle. f) Representative images and g) quantification of pericyte migration in the scratch assay. n = 3, two-way ANOVA. Scale bar = 500 µm. * - $p < 0.05$ vs vehicle control, # - $p < 0.05$ vs PDGF-BB-treated.

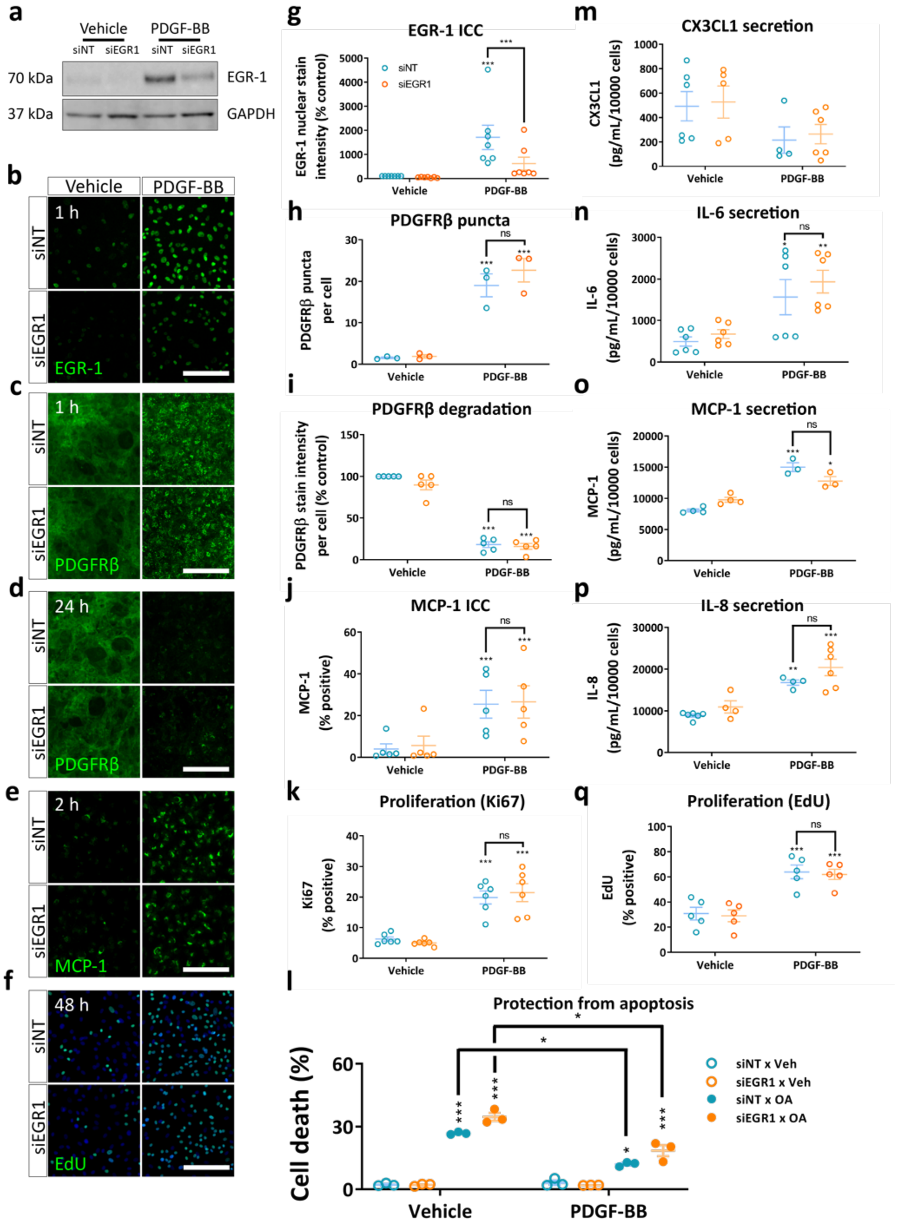

Supplementary Figure 5: EGR-1 does not affect PDGF-BB-induced changes to PDGFRβ dynamics, secretion, proliferation, or survival in pericytes. Pericytes were treated with siRNA against EGR-1 or non-targeted siRNA (50 nM) for 96 hours, then treated with PDGF-BB (10 ng/mL) for up to 48 h, and were fixed and immunostained, lysates taken for western blot, or conditioned media taken for cytometric bead array. a) Western blot against EGR-1 and GAPDH loading control in pericytes treated with PDGF-BB (1 h) with or without siEGR1. Representative images and quantification of b, g) EGR-1 immunostaining, c, h) PDGFRβ puncta formation, d, i) PDGFRβ degradation, e, j) MCP-1 immunostaining, f, q) EdU incorporation, and k) Ki67 immunostaining in pericytes treated with PDGF-BB (10 ng/mL) with or without siEGR1. Scale bar = 200 µm. Secretion of m) CX3CL1, n) IL-6, o) MCP-1, and p) IL-8 in response to PDGF-BB treatment with or without siEGR1. Pericytes were treated with siRNA against EGR-1 or non-targeted siRNA (50 nM) for 96 hours, then treated with PDGF-BB (10 ng/mL) for 24 h, and okadaic acid (50 nM) for a further 24 h and viability assessed by the ReadyProbes^{TM} viability imaging kit. l) Protection of pericytes from to OA-induced apoptosis by PDGF-BB in cells treated with or without siEGR1. n = 3-6, two-way ANOVA. * - $p < 0.05$, ** - $p < 0.01$, *** - $p < 0.001$ vs vehicle control, # - $p < 0.05$, ## - $p < 0.01$, ### - $p < 0.001$ vs PDGF-BB-treated.

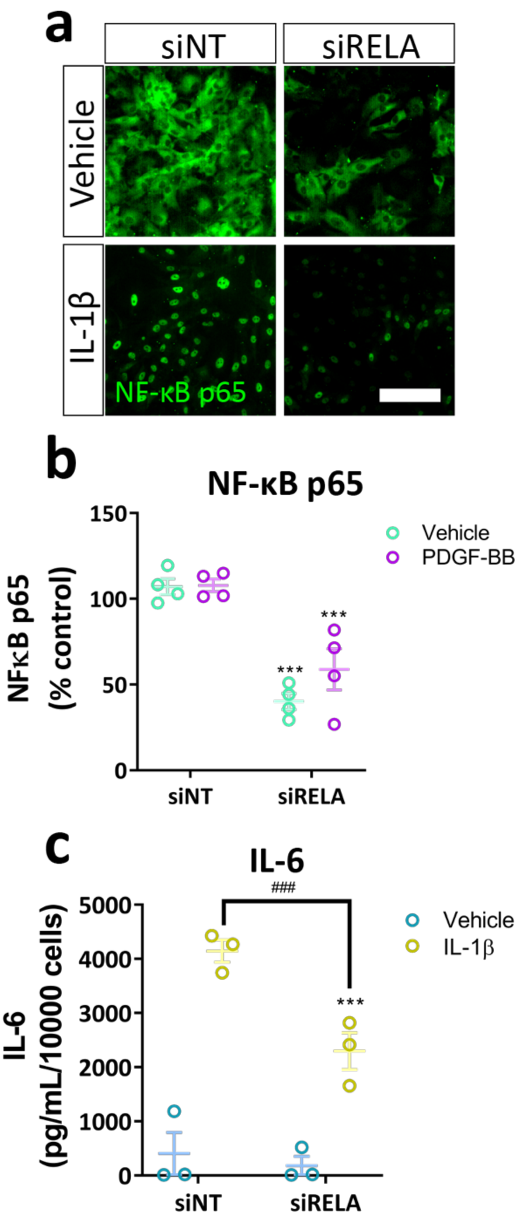

Supplementary Figure 6: Validation of NF-κB p65 knockdown. Pericytes were treated with siRNA against NF-κB p65 (siRELA; 50 nM) or non-targeted siRNA for 96 hours. Cells were then treated with PDGF-BB (10 ng/mL) or IL-1β (10 ng/mL), and fixed and immunostained, or conditioned media harvested for cytometic bead array. a) Representative images and b) quantification of NF-κB expression in pericytes treated with siRELA, with or without IL-1β and PDGF-BB. c) Secretion of IL-6 in pericytes treated with siRELA, with or without IL-1β. n = 3-4, two-way ANOVA. *** - $p < 0.05$ vs vehicle control, ### - $p < 0.05$ vs PDGF-BB-treated.

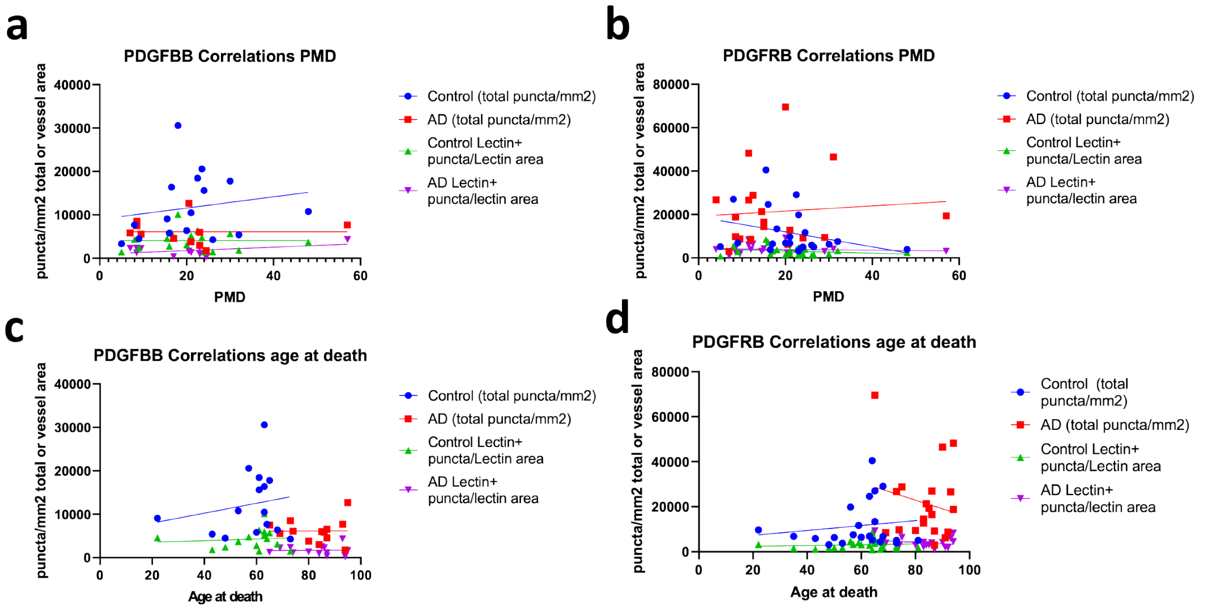

Supplementary Figure 7: No correlation between age or post-mortem delay of patient samples and PDGFB or PDGFRB levels measured by RNAscope in Figure 1g. a) Regression between *PDGFB* and post-mortem delay (PMD) in control and AD samples for the total number of puncta measured and the number of puncta normalised to lectin area. b) Regression between *PDGFRB* and PMD in control and AD samples for the total number of puncta measured and the number of puncta normalised to lectin area. c) Regression between *PDGFB* and age at death in control and AD samples for the total number of puncta measured and the number of puncta normalised to lectin area. d) Regression between *PDGFRB* and age at death in control and AD samples for the total number of puncta measured and the number of puncta normalised to lectin area.

Figure 3

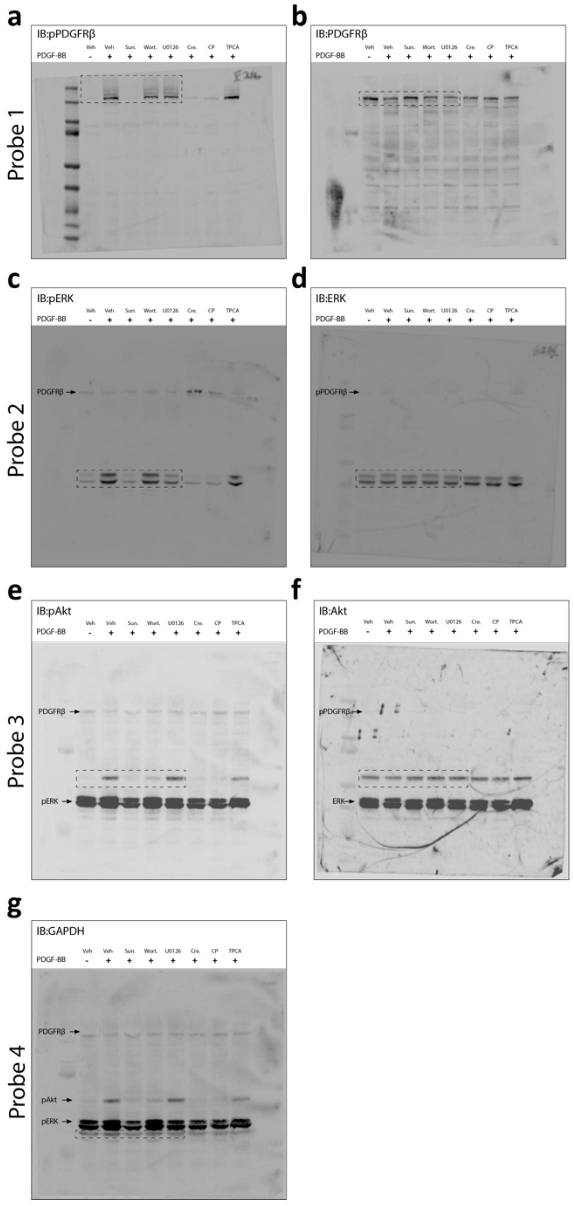

Figure S5

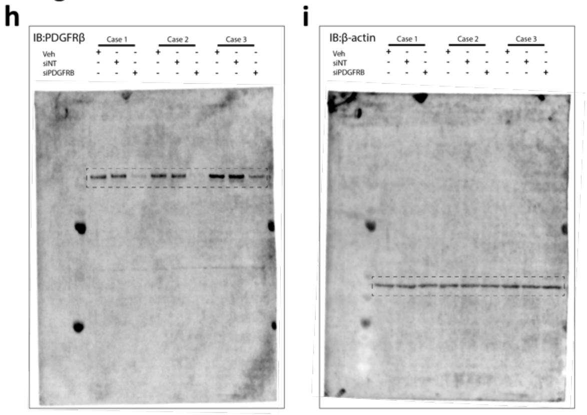

Figure S8

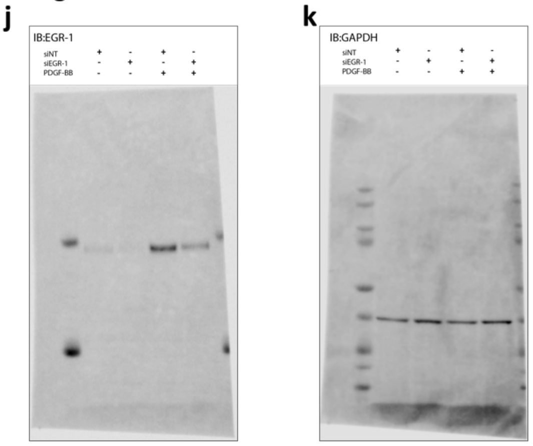

Supplementary Figure 8: Full images of blots used to provide representative cropped bands. Pericytes were treated as in figure 4, and sequentially blotted for a, b) phosphorylated and total PDGFRβ, c, d) ERK, and e, f) Akt then g) loading control GAPDH. Pericytes were treated as in supplementary figure 2, and blotted for h) PDGFRβ and i) loading control β-actin. Pericytes were treated as in supplementary figure 4, then immunoblotted for j) EGR-1 and k) GAPDH.

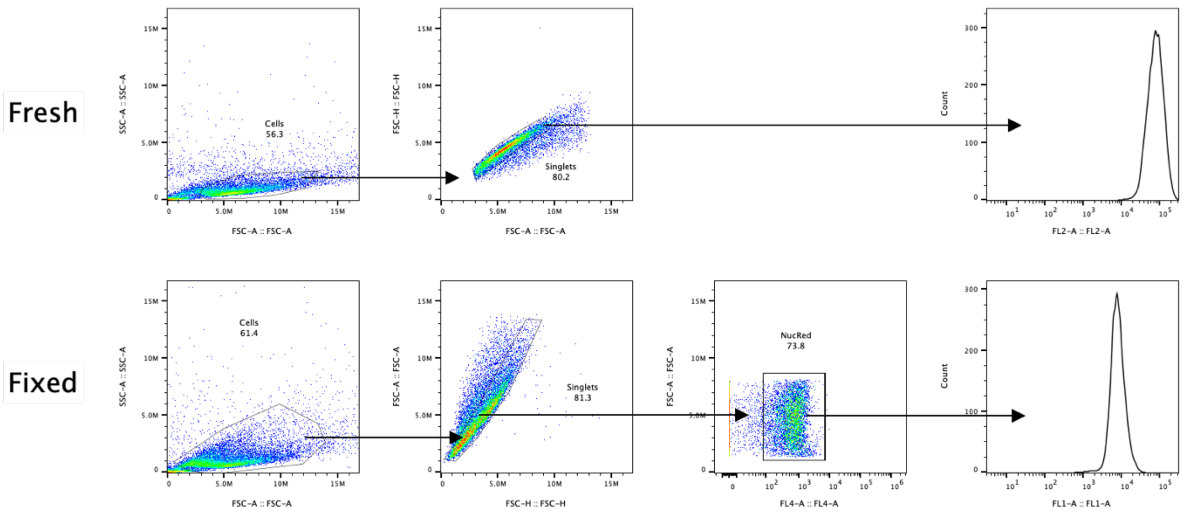

Supplementary Figure 9: Representative gating strategy for PDGFRB surface and total expression in live and fixed cells.

Supplementary Table 1: Case details of tissue used in tissue microarray and immunohistochemistry experiments. COD = cause of death, MTG = middle temporal gyrus, NN = neurologically normal, AD = Alzheimer's disease, PMD = post-mortem delay.

Case	Region	Age	Pathology	Sex	PMD (h)	Notes
**02F/393**	**MTG**	87	NN	F	11	
**4680**	**MTG**	80	NN	M	12	
**4734**	**MTG**	79	NN	M	8	
**6013**	**MTG**	69	NN	F	11.5	
**H121**	**MTG**	64	NN	F	5	
**H122**	**MTG**	72	NN	F	9	
**H123**	**MTG**	78	NN	M	13	
**H127**	**MTG**	59	NN	F	21	
**H136**	**MTG**	75	NN	M	13	
**H137**	**MTG**	77	NN	M	12	
**H139**	**MTG**	73	NN	M	5.5	
**H144**	**MTG**	76	NN	M	19	
**H145**	**MTG**	54	NN	M	8	
**H148**	**MTG**	64	NN	M	7	
**H150**	**MTG**	78	NN	M	11	
**H151**	**MTG**	64	NN	F	5	COD: coronary atherosclerosis. Non-specific diffuse beta-amyloid plaques = age related; No AD; No cortical or LB; CN & CB unremarkable.
**H152**	**MTG**	79	NN	M	18	
**H153**	**MTG**	76	NN	M	8	
**H155**	**MTG**	61	NN	M	7	
**H156**	**MTG**	89	NN	M	19	
**H159**	**MTG**	53	NN	M	16.5	COD: ischemic heart disease. Control specimen, normal for age.
**H160**	**MTG**	77	NN	M	23	
**H164**	**MTG**	73	NN	M	13	
**H165**	**MTG**	43	NN	F	26	COD: nitrogen poisoning. No significant histological abnormalities.
**H167**	**MTG**	51	NN	M	23.5	COD: asphyxia. Hepatitis C positive.
**H168**	**MTG**	63	NN	M	9	COD: ischemic heart disease. No significant histological abnormalities.
**H169**	**MTG**	81	NN	M	24	COD: asphyxia. No LBD; low AD change (A2 B0 C1); cerebral amyloid angiopathy; Tau+ grain-like path in CA1.
**H170**	**MTG**	60	NN	M	17	COD: ischemic heart disease. No significant histological abnormalities.
**H174**	**MTG**	59	NN	M	24.5	COD: aortic aneurysm. No significant histological changes.
**H177**	**MTG**	22	NN	M	21	COD: asphyxia. Control specimen: No significant pathological changes.
**H180**	**MTG**	73	NN	M	33	
**H183**	**MTG**	61	NN	M	13	COD: multiple organ failure. Control specimen: No significant pathological changes.
**H184**	**MTG**	35	NN	M	20	COD: electrocution. Control specimen: No significant pathological changes.
**H185**	**MTG**	50	NN	M	28	COD: obscure causes. Control specimen: No significant pathological changes.
**H186**	**MTG**	68	NN	M	21	COD: ischemic heart disease. Control specimen: No significant pathological changes.
**H187**	**MTG**	98	NN	F	15	
**H188**	**MTG**	83	NN	M	17	
**H190**	**MTG**	72	NN	F	19	
**H191**	**MTG**	77	NN	M	20	

**H192**	**MTG**	65	NN	F	23	COD: ischemic heart disease. No significant histological changes, Hepatitis B44 positive.
**H194**	**MTG**	68	NN	M	22.5	COD: coronary atherosclerosis. No significant histological abnormalities.
**H195**	**MTG**	65	NN	M	18	COD: ischemic heart disease. No significant histological abnormalities.
**H196**	**MTG**	85	NN	F	15	
**H198**	**MTG**	67	NN	F	27	
**H200**	**MTG**	56	NN	M	23	COD: asphyxia. No significant histological abnormalities.
**H202**	**MTG**	83	NN	M	14	
**H209**	**MTG**	48	NN	M	23	COD: ischemic heart disease. Appearances unremarkable for age.
**H215**	**MTG**	67	NN	F	23.5	COD: ischemic heart disease. Control specimen: no significant histological abnormalities.
**H226**	**MTG**	73	NN	F	48	COD: mesothelioma. Cerebral age-related change.
**H230**	**MTG**	57	NN	F	32	COD: carcinomatosis (renal). Age-related cerebral changes.
**H231**	**MTG**	65	NN	M	8	COD: ischemic heart disease. No significant histological abnormalities.
**H238**	**MTG**	63	NN	F	16	COD: dissecting aortic aneurysm. No significant histological abnormalities.
**H239**	**MTG**	64	NN	M	15.5	COD: ischemic heart disease. No significant histological abnormalities.
**H240**	**MTG**	73	NN	M	26.5	COD: ruptured aneurysm, abdominal haemorrhage. Mild cerebral cortical abnormalities. Sparse neurofibrillary tangles.
**H242**	**MTG**	61	NN	M	19.5	COD: coronary atherosclerosis. Relatively unremarkable; Diffuse beta amyloid plaques in MTG, cerebral amyloid angiopathy.
**H245**	**MTG**	63	NN	M	20	COD: asphyxia. No significant abnormality identified.
**Avg**		67.4		28.6	17.5	
**AZ33**	**MTG**	65	AD	M	20	COD: hypostatic pneumonia. CERAD: Definite Alzheimer's disease. Atrophy: mid-1, Tangles: mild-1, Plaques: mod-2, ARP: C.
**AZ34**	**MTG**	74	AD	F	18	ARP: C.
**AZ37**	**MTG**	83	AD	M	4	ARP: B.
**AZ38**	**MTG**	80	AD	M	5.5	ARP: C.
**AZ39**	**MTG**	74	AD	M	12	ARP: C.
**AZ42**	**MTG**	60	AD	M	7	ARP: C.
**AZ43**	**MTG**	80	AD	M	21	ARP: B.
**AZ45**	**MTG**	82	AD	M	4.5	ARP: B.
**AZ46**	**MTG**	82	AD	F	22	ARP: B.
**AZ52**	**MTG**	68	AD	F	36	ARP: C.
**AZ55**	**MTG**	51	AD	M	4	ARP: B.
**AZ57**	**MTG**	82	AD	F	14.5	ARP: A.
**AZ58**	**MTG**	75	AD	M	20	ARP: C.
**AZ59**	**MTG**	83	AD	M	15	ARP: A.
**AZ61**	**MTG**	87	AD	F	7.5	ARP: C.
**AZ64**	**MTG**	67	AD	M	8	ARP: C.
**AZ65**	**MTG**	77	AD	F	16	ARP: C.
**AZ68**	**MTG**	68	AD	F	7	ARP: C.
**AZ71**	**MTG**	62	AD	F	6	ARP: C.
**AZ72**	**MTG**	70	AD	F	7	Braak: V, ARP: C.

**AZ73***	**MTG**	87	AD	F	14.5	Mixed Pathology: Alzheimer's disease & Cortical Lewy Body Disease Braak: IV, Atrophy: 1/3, Tangles: 2/3, Plaques: 2/3, ARP: B
**AZ74**	**MTG**	85	AD	F	16	Braak: VI, ARP: C.
**AZ75**	**MTG**	85	AD	M	25	Braak: V/VI, ARP: C.
**AZ77**	**MTG**	81	AD	F	16	Braak: IV/VI, ARP: B.
**AZ78**	**MTG**	87	AD	F	7	COD: general inanition, dementia. CERAD: probable Alzheimer's disease. Braak: 3/6, Atrophy: 2/3, Tangles: 2/3, Plaques: 1/3, ARP: B.
**AZ80**	**MTG**	77	AD	M	4.5	Braak: IV/IV, ARP: C.
**AZ81**	**MTG**	82	AD	F	18	Braak: IV/IV, ARP: C.
**AZ82**	**MTG**	80	AD	F	18	Braak: IV/IV, ARP: C.
**AZ83**	**MTG**	60	AD	F	16	Braak: IV/IV, ARP: C.
**AZ85**	**MTG**	85	AD	M	57	COD: AD, prostate cancer. CERAD: probable Alzheimer's disease. Braak: 4/6, Atrophy: 0/3, Tangles: 2/3, Plaques: 1/3, ARP: B.
**AZ86**	**MTG**	92	AD	M	8.5	COD: bronchopneumonia, chronic renal failure. CERAD: possible Alzheimer's disease. Braak: 3/6, Atrophy: 0/3, Tangles: 1/3, Plaques: 1/3, ARP: A.
**AZ87**	**MTG**	73	AD	M	5	
**AZ88**	**MTG**	83	AD	M	21	COD: pneumonia. CERAD: Definite Alzheimer's disease. Braak: 4/6; Atrophy: 2/3, Tangles: 3/3, Plaques: 3/3, ARP: C.
**AZ89**	**MTG**	80	AD	F	25	COD: advanced dementia. CERAD: Definite Alzheimer's disease. Braak: 6/6; Atrophy: 3/3, Tangles: 3/3, Plaques: 3/3, ARP: C.
**AZ90**	**MTG**	73	AD	M	4	COD: gastrointestinal haemorrhage. CERAD: Definite Alzheimer's disease. Braak: 4/6; Atrophy: 3/3, Tangles: 3/3, Plaques: 3/3, ARP: C.
**AZ91**	**MTG**	80	AD	M	29	COD: sepsis, aspiration pneumonia. CERAD: Definite Alzheimer's disease. Braak: 5/6; Atrophy: 2/3, Tangles: 3/3, Plaques: 3/3, ARP: C.
**AZ92**	**MTG**	93	AD	F	11.5	COD: bronchopneumonia. CERAD: Probable Alzheimer's disease. Braak: 4/6, Atrophy: 3/3, Tangles: 3/3, Plaques: 3/3, ARP: B.
**AZ93**	**MTG**	83	AD	M	15	COD: AD dementia. CERAD: Probable Alzeimer Disease. Mod plaque density; Braak Stage V.
**AZ95**	**MTG**	69	AD	M	12	COD: bronchopneumonia, aspiration pneumonia. CERAD: Alzheimer's disease. Braak: 5/6, Atrophy: 3/3, Tangles: 2/3, Plaques: 3/3, ARP: C.
**AZ96**	**MTG**	74	AD	F	8.5	COD: metastatic cancer, likely gastric. CERAD: Alzheimer's disease Braak: 5/6; Atrophy: 3/3, Tangles: 3/3, Plaques: 3/3, ARP: C.
**AZ98**	**MTG**	91	AD	F	20.5	COD: Alzheimer's dementia, atrial fibrillation. Alzheimer's disease.
**AZ99**	**MTG**	94	AD	F	8.5	COD: multiple organ systems failure. Alzheimer's-type neuropath. change (A3, B3, C2), Braak V-VI; small vessel cerebrovascular wth focal lacunar

						infarction; focal cerebral amyloid angiopathy.
**AZ101**	**MTG**	75	AD	M	12.5	COD: right lower lobe pneumonia. AD neuopathology. change (A3, B3, C2), Braak VI; cerebral amyloid angiopathy; old infarction in R temporo-occipital; small vessel cerebrovascular disease (CT).
**AZ102**	**MTG**	84	AD	F	14.5	COD: lower respiratory tract infection. Alzheimer's-type neuropathological change (A3, B2, C2), Braak IV; hyaline arteriosclerosis.
**AZ103**	**MTG**	87	AD	M	<24	COD: cerebrovascular event. Alz-type neuropathologic change (A3, B2, C2), Braak IV; cerebral amyloid angiopathy; small vessel disease.
**AZ107**	**MTG**	86	AD	M		COD: chest infection. Intermediate AD change (A2, B3, C1), Braak VI; Amygdala predominant Lewy body disease; deep small vessel disease; cerebral amyloid angiopathy.
**AZ108**	**MTG**	94	AD	F	11.5	COD: Alzheimer's disease. Consistent with Alzheimer's disease (NIA-AA score A3 B3 C2, high degree of AD change), LBD, amygdala predominant, cerebral amyloid angiopathy, hyaline arteriolosclerosis.
**AZ109**	**MTG**	90	AD	F	31	COD: end stage dementia. Consistent with Alzheimer's disease (NIA-AA score A3 B2 C1, intermediate AD change), cerebral amyloid angiopathy, hyaline arteriolosclerosis.
**AZ110**	**MTG**	86	AD	F	15	COD: severe dementia. Consistent with Alzheimer's disease (NIA-AA score A3 B3 C2, high AD change); hippocampal sclerosis with associated TDP-43 path; LBD, diffuse; focal cerebral amyloid angiopathy; small vessel disease.
**Avg**		83.4		47.6	17.5	
**E204**	**MTG**	45	Epilepsy	F	0.5	Epilepsy, hippocampal sclerosis ILAE type 2.

Supplementary Table 2: Case details of pericyte lines used for *in vitro* experiments. MTG = middle temporal gyrus, POD = post-operative delay, PMD = post-mortem delay, NN = neurologically normal, AD = Alzheimer's disease, CLBD = cortical Lewy body disease.

**Case**	**Region**	**Age**	**Pathology**	**Sex**	**POD/PMD (h)**	**Notes**
**E203**	**MTG**	46	Epilepsy	F	0.5	Epilepsy, grade 1 mesial temporal sclerosis.
**E204**	**MTG**	45	Epilepsy	F	0.5	Epilepsy, hippocampal sclerosis ILAE type 2.
**E206**	**MTG**	29	Epilepsy	F	0.5	Epilepsy, grade 3 mesial temporal sclerosis.
**E208**	**MTG**	52	Epilepsy	F	0.5	Left temporal lobe and hippocampus. Moderate to marked mesial temporal sclerosis (grade 3).
**E213**	**MTG**	23	Epilepsy	M	0.5	Left Anterior Temporal Lobe: moderate increase in neurons in temporal WM; some patchy mild astrocytic gliosis in temporal WM on GFAP stain; no features to suggest cortical dysplasia or malformation of cortical development. No evidence of neoplasma. Hippocampus:

**E215**	**MTG**	29	Epilepsy	F	0.5	No posterior hippocampus identifable - inadequate to confirm diagnosis of hippocampal sclerosis; no neoplasma seen. Diagnosis: 1.2.3. Right anterior temporal lobe, posterior hippocampus head of hippocampus. Moderate to marked mesial temporal sclerosis (grade 3).
**Avg**		37.3		83.3	0.5	
**H189**	**MTG**		NN	M	16	COD: asphyxia. Control specimen: No significant pathological changes.
**H204**	**MTG**	66	NN	M	9	COD: Ischaemic heart disease; no significant histological abnormalities.
**H242**	**MTG**	61	NN	M	19.5	COD: Coronary atherosclerosis; Generally unremarkable; diffuse beta amyloid plaques in MTG, cerebral amyloid angiopathy.
**Avg**		63.5		0	13.5	

Supplementary Table 3: Case details of endothelial lines used for *in vitro* experiments. MTG = middle temporal gyrus, POD = post-operative delay.

**Case**	**Region**	**Age**	**Pathology**	**Sex**	**POD (h)**	**Notes**
**E213**	**MTG, hippocampus**	23	Epilepsy	M	0.5	Epilepsy, patchy gliosis, no cortical dysplasia, no neoplasia, presumed hippocampal sclerosis.
**E215**	**MTG**	29	Epilepsy	F	0.5	Right anterior temporal lobe, posterior hippocampus head of hippocampus. Moderate to marked mesial temporal sclerosis (grade 3).
**T126**	**Occipital cortex**	46	Tumour (melanoma met)	M	0	Endothelia cultured from occipital cortex overlying metastasis.
**T131**	**Cerebellum**	20	Tumour	M	0	Pilocytic astrocytoma grade I.
**T138**	**Frontal Lobe**	30	Tumour	M	0	Left frontal oligodendroglioma grade II.
**SS32**	**Right parietal**	11	Cortical dysplasia	F	0	None.
**SS37**	**Left cortical**	9	Cortical dysplasia	M	0.5	None.
**SS42**	**Right temporal lobe, hippocampus**	8	Cortical dysplasia	M	1	None.
**Avg**		22.0		25.0	0.3	

Supplementary Table 4: Details of antibodies used for these studies.

Antigen	Species	Company	Catalogue	ICC	IHC	WB	Flow
**Primary antibody (clone)**							
**β-actin (AC-15)**	Rabbit	Abcam	ab6276	-	-	1:2000	-
**Akt (40D4)**	Mouse	Cell Signalling	2920	-	-	1:2000	-
**pAkt (poly)**	Rabbit	Cell Signalling	9271	-	-	1:1000	-
**cJun (poly)**	Rabbit	Santa Cruz	sc-1694	1:500	-	-	-
**EEA-1 (poly)**	Rabbit	Abcam	ab2900	1:10000	-	-	-
**EGR-1 (poly)**	Rabbit	Santa Cruz	sc-198	1:500	-	-	-
**EGR-1 (15F7)**	Rabbit	Cell Signalling	4153	-	-	1:500	-
**ERK1/2 (L34F12)**	Mouse	Cell Signalling	4696	-	-	1:500	-
**pERK1/2 (D13.14.4E)**	Rabbit	Cell Signalling	4370	-	-	1:500	-
**pERK1/2 (poly)**	Rabbit	Cell Signalling	9101	1:1000	-	-	-
**GAPDH (Abcam 9484)**	Mouse	Abcam	ab9484	-	-	1:2000	-
**IgG2a-κ control (G155-178)**	Mouse	BD Pharmingen	555571	-	-	-	1:20
**IL-6 (poly)**	Goat	R&D	AF-206	1:2500	-	-	-
**Ki67 (MIB-1)**	Mouse	Dako	M7240	1:500	-	-	-
**LAMP-1 (H4A3)**	Mouse	DSHB	AB_2296838	1:500	-	-	-
**MCP-1 (poly)**	Rabbit	Abcam	ab9669	1:500	-	-	-
**NF-κB p65 (poly)**	Rabbit	Santa Cruz	sc-372	1:500	-	-	-
**NF-κB p65 (F-6)**	Mouse	Santa Cruz	sc-8008	1:500	-	-	-
**PDGFRβ (BR7212)**	Mouse	BioRad	7460-3104	1:500	-	-	1:500
**PDGFRβ (Y92)**	Rabbit	Abcam	ab32570	1:500	1:100	-	-
**PDGFRβ (poly)**	Goat	R&D	AF385	1:1000	-	1:1000	-
**PDGFRβ-PE (28D4)**	Mouse	BD Pharmingen	558820	-	-	-	1:20
**pPDGFRβ (C63G6)**	Rabbit	Cell Signalling	4549	-	-	1:1000	-
**Rab5 (poly)**	Rabbit	Abcam	ab18211	1:500	-	-	-
**Rab7 (EPR7589)**	Rabbit	Abcam	ab137029	1:500	-	-	-
**SMAD2/3 (C-8)**	Mouse	Santa Cruz	sc-133098	1:500	-	-	-
**STAT1 (D1K9Y)**	Rabbit	Cell Signalling	14994	1:500	-	-	-
**STAT3 (124H6)**	Mouse	Cell Signalling	9139	1:500	-	-	-
**Biotinylated UEA lectin**	-	Vector Labs	B-1065	-	1:1000	-	-
**Secondary antibodies**							
**Anti-mouse Alexa 488**	Goat	Life Tech	A11001	1:500	1:250	-	1:500
**Anti-mouse Alexa 594**	Goat	Life Tech	A11005	1:500	1:250	-	-
**Anti-mouse Alexa 647**	Goat	Life Tech	A21235	1:500	-	-	-
**Anti-mouse Alexa 488**	Donkey	Life Tech	A21202	1:500	1:250	-	-
**Anti-rabbit Alexa 488**	Goat	Life Tech	A11008	1:500	1:250	-	-
**Anti-rabbit Alexa 594**	Goat	Life Tech	A11012	1:500	1:250	-	-
**Anti-rabbit Alexa 647**	Goat	Life Tech	A27040	1:500	1:250	-	-
**Anti-rabbit Alexa 594**	Donkey	Life Tech	A21207	1:500	-	-	-
**Anti-rabbit Alexa 647**	Donkey	Life Tech	A31573	-	1:250	-	-
**Anti-goat Alexa 488**	Donkey	Life Tech	A11055	1:500	-	-	-
**Anti-goat Alexa 647**	Donkey	Life Tech	A21447	1:500	-	-	-
**Anti-mouse IRDye-680LT**	Goat	LiCOR	926-68020	-	-	1:10000	-
**Anti-rabbit IRDye-800CW**	Goat	LiCOR	926-32211	-	-	1:10000	-
**Anti-rabbit IRDye-800CW**	Donkey	LiCOR	925-32213	-	-	1:10000	-
**Anti-goat IRDye-680LT**	Donkey	LiCOR	926-68024	-	-	1:10000	-
**Streptavidin-Cy5**	-	Jackson IR	016-160-074	-	1:250	-	-

Supplementary Table 5: Cytometric bead array targets and positions.

Antibody	Cat no.	Bead position
**Fractalkine (CX3CL1)**	560265	C6
**IL-6 (interleukin-6)**	558276	A7
**IL-8 (interleukin-8)**	558277	A9
**MCP-1 (monocyte chemoattractant protein-1)**	558287	D8

Supplementary Table 6: Targets and sequences for primers used in this study.

Accession	Gene (protein)	Sequence (5' to 3')		Amplicon (bp)
**NM_001150.3**	*ANPEP*	Fw	ACCTGGGTGCTGACTATGCGGA	113
	(CD13) [1]	Rv	ACTGCCATCACGCGGTACACA	
**NM_002982.3**	*CCL2*	Fw	CAGCCAGATGCAATCAATGCC	90
	(MCP-1) [2,3]	Rv	TGGAATCCTGAACCCACTTCT	
**NM_002996.4**	*CX3CL1*	Fw	ATTCTTTCCTGAGGCTGGGC	74
	(CX3CL1) [3]	Rv	GGTCTTGGAGGGCAGAGAAC	
**NM_000584.3**	*CXCL8*	Fw	CAGAGACAGCAGAGCACACA	70
	(IL-8) [2,3]	Rv	GTGAGATGGTTCCTTCCGGT	
**NM_058229.3**	*FBXO32*	Fw	TCTCCCCATCCGTCTAGTCC	85
	(FBX32)	Rv	GTCTTCACCCAGTTCTGCCC	
**NM_002046.4**	*GAPDH*	Fw	CATGAGAAGTATGACAACAGCCT	113
	(GAPDH) [1,2,4]	Rv	AGTCCTTCCACGATACCAAAGT	
**NM_000600.3**	*IL6*	Fw	GGCTGCAGGACATGACAACT	102
	(IL-6) [3]	Rv	ATCTGAGGTGCCCATGCTAC	
**NM_001145966.1**	*MKI67*	Fw	TTGGGGTTCGCTCTTGGATC	90
	(Ki67) [5]	Rv	GTGGCATTGTCTAGGGCAGT	
**NM_002421.3**	*MMP1*	Fw	GCCATCACTTACCTTGCACTG	94
	(MMP-1)	Rv	GAGACACCACACCCCAGAAC	
**NM_000442.4**	*PECAM1*	Fw	AAAGCTGTCCCTGATGCCGT	80
	(CD31) [6]	Rv	TCTGGCCTTGCTGTCTAAGTTC	
**NM_002608.3**	*PDGFB*	Fw	GATACTTTGCGCGCACACAC	91
	(PDGF-B)	Rv	GGTTTTCTCTTTGCAGCGAGG	
**NM_002609.3**	*PDGFRB*	Fw	CGCAAAGAAAGTGGGCGGCT	101
	(PDGFRβ) [1,4]	Rv	TGCAGGATGGAGCGGATGTGGT	

Supplementary Table 7: Cases used for NanoString analysis (Figure 1g).

Case	Region	Age	Pathology	Sex	POD/PMD (h)	Notes
**H121**	**MFG**	64	NN	F	6.5	COD: pulmonary embolism. No significant histological abnormalities
**H122**	**MFG**	72	NN	F	9	COD: emphysema. All unremarkable except hippocampus with agonal haemorrhages; ok as control
**H127**	**MFG**	59	NN	F	21	COD: pulmonary embolism. No Alzheimer's disease change (A0 B0 C0); No transitional or Lewy body disease
**H129**	**MFG**	48	NN	M	12	COD: pulmonary embolism. No significant cerebral pathology
**H131**	**MFG**	73	NN	F	13	COD: ischemic heart disease. No significant histological abnormalities
**H165**	**MFG**	43	NN	F	26	COD: nitrogen poisoning. No significant histological abnormalities
**H170**	**MFG**	60	NN	M	17	COD: ischemic heart disease. No significant histological abnormalities
**H202**	**MFG**	83	NN	M	14	COD: ruptured abdominal aortic aneurysm. No significant changes of degenerative type found.

		62.8		62.5	14.8	
**AZ43**	**MFG**	80	D	M	21	COD: bronchopneumonia. CERAD: Probable Alzheimer's disease. Atrophy: mild-1, Tangles: mod-2, Plaques: mod-2, ARP: B
**AZ45**	**MFG**	82	AD	M	4.5	COD: pneumonia, stroke (6 wks). CERAD: Probable Alzheimer's disease. Atrophy: mild-1, Tangles: mod-2, Plaques: mod-2, ARP: B
**AZ65**	**MFG**	77	AD	F	16	COD: bronchopneumonia. CERAD: Alzheimer's disease (definite). Atrophy: severe, Tangles: mod, Plaques: numerous, ARP: C
**AZ71**	**MFG**	61	AD	F	6	COD: severe dementia. CERAD: Definite Alzheimer's disease. Braak: VI; Atrophy: 2/3, Tangles: 3/3, Plaques: 3/3, ARP: C
**AZ72**	**MFG**	70	AD	F	7	COD: lung cancer. CERAD: Indicative of Alzheimer's disease. Braak: V; Atrophy: 0/3, Tangles: 1/3, Plaques: 3/3, ARP: C
**AZ80**	**MFG**	77	AD	M	4.5	COD: myocardial infarction. CERAD: Definite Alzheimer's disease. Braak: VI; Atrophy: 3/3, Tangles: 3/3, Plaques: 3/3, ARP: C
**AZ84**	**MFG**	82	AD	M	18.5	COD: bronchopneumonia. CERAD: probable Alzheimer's disease; Mild Cortical Lewy Body disease. Braak: III, Atrophy: 1/3, Tangles: 1/3, Plaques: 1/3, ARP: A
**AZ86**	**MFG**	92	AD	M	8.5	COD: bronchopneumonia, chronic renal failure. CERAD: possible Alzheimer's disease. Braak: III, Atrophy: 0/3, Tangles: 1/3, Plaques: 1/3, ARP: A
**Avg**		77.6		37.5	10.75	

Supplementary Table 8: Correlations between post-mortem delay (PMD), age at death, and *PDGFB*/*PDGFRB* signal detected by *in situ* hybridisation.

Gene	Condition	Covariate	Measurement	R^2	p-value
PDGFB	Control	PMD	Total puncta	0.032	0.508
	AD	PMD	Total puncta	0.000	0.999
	Control	Age at death	Total puncta	0.036	0.500
	AD	Age at death	Total puncta	0.000	0.97
	Control	PMD	Puncta/Lectin area	0.000	0.992
	AD	PMD	Puncta/Lectin area	0.230	0.135
	Control	Age at death	Puncta/Lectin area	0.011	0.713
	AD	Age at death	Puncta/Lectin area	0.000	0.920
PDGFRB	Control	PMD	Total puncta	0.093	0.168
	AD	PMD	Total puncta	0.001	0.734
	Control	Age at death	Total puncta	0.020	0.526
	AD	Age at death	Total puncta	0.038	0.408
	Control	PMD	Puncta/Lectin area	0.051	0.312
	AD	PMD	Puncta/Lectin area	0.010	0.679
	Control	Age at death	Puncta/Lectin area	0.015	0.593
	AD	Age at death	Puncta/Lectin area	0.054	0.325
