# Supplementary material for: Characterisation of PDGF-BB:PDGFRβ signalling pathways in human brain pericytes: evidence of disruption in Alzheimer’s disease
Source: Commun Biol. 2022 Mar 17;5:235. doi: 10.1038/s42003-022-03180-8 (PMC8931009; doi:10.1038/s42003-022-03180-8)
Supplement: Supplementary file 2 — Description of Additional Supplementary Files [file 42003_2022_3180_MOESM2_ESM.pdf]

Description of Additional Supplementary Files

File name: Supplementary Data
Description: Source data.
