# Supplementary material for: Characterisation of PDGF-BB:PDGFRβ signalling pathways in human brain pericytes: evidence of disruption in Alzheimer’s disease
Source: Commun Biol. 2022 Mar 17;5:235. doi: 10.1038/s42003-022-03180-8 (PMC8931009; doi:10.1038/s42003-022-03180-8)
Supplement: Supplementary file 4 — Reporting Summary [file 42003_2022_3180_MOESM4_ESM.pdf]

nature portfolio

Corresponding author(s): Mike Dragunow

Last updated by author(s): Aug 10, 2021

Reporting Summary

Nature Portfolio wishes to improve the reproducibility of the work that we publish. This form provides structure for consistency and transparency in reporting. For further information on Nature Portfolio policies, see our Editorial Policies and the Editorial Policy Checklist.

Statistics

For all statistical analyses, confirm that the following items are present in the figure legend, table legend, main text, or Methods section.

n/a	Confirmed	
☐	☒	The exact sample size (*n*) for each experimental group/condition, given as a discrete number and unit of measurement
☒	☐	A statement on whether measurements were taken from distinct samples or whether the same sample was measured repeatedly
☐	☒	The statistical test(s) used AND whether they are one- or two-sided<br>*Only common tests should be described solely by name; describe more complex techniques in the Methods section.*
☒	☐	A description of all covariates tested
☐	☒	A description of any assumptions or corrections, such as tests of normality and adjustment for multiple comparisons
☐	☒	A full description of the statistical parameters including central tendency (e.g. means) or other basic estimates (e.g. regression coefficient) AND variation (e.g. standard deviation) or associated estimates of uncertainty (e.g. confidence intervals)
☒	☐	For null hypothesis testing, the test statistic (e.g. *F*, *t*, *r*) with confidence intervals, effect sizes, degrees of freedom and *P* value noted<br>*Give P values as exact values whenever suitable.*
☒	☐	For Bayesian analysis, information on the choice of priors and Markov chain Monte Carlo settings
☒	☐	For hierarchical and complex designs, identification of the appropriate level for tests and full reporting of outcomes
☒	☐	Estimates of effect sizes (e.g. Cohen's *d*, Pearson's *r*), indicating how they were calculated

Our web collection on statistics for biologists contains articles on many of the points above.

Software and code

Policy information about availability of computer code

Data collection	Cells were imaged using the ImageXpress® Micro XLS automated fluorescent microscope. Proteome profilers were imaged with LI-COR Odyssey® FC imaging system. Cytometric bead arrays were performed on an Accuri C6 Flow Cytometer (BD Biosciences). Human tissue microarray sections were imaged with Metasystems V-slide scanning microscope, and confocal recording done using an FV1000 confocal microscope (Olympus). RNAseq data were collected on a Novaseq 6000.
Data analysis	Image analysis was performed using MetaXpress® software (Molecular Devices). Cytometric bead array analysis was performed using FCAP Array Software. Proteome profilers were quantified with Image Studio Software. Prism (Graphpad Software) was used for statistical testing and data are presented as mean +/- S.E.M. Multiple comparisons are noted in the corresponding figure legends. Analysis of RNA-seq data utilised the pipeline developed by Pertea et al, 2016 with HISAT2 (v2.1.0), StringTie (v1.3.4) and Ballgown (R version 3.5.1). Differentially expressed genes were identified as having FPKM > 8 in one group, adjusted p-value < 0.05, and log2 fold change greater than 2. Heatmaps from RNAseq were made in RStudio with gplots, heatmap.2.

For manuscripts utilizing custom algorithms or software that are central to the research but not yet described in published literature, software must be made available to editors and reviewers. We strongly encourage code deposition in a community repository (e.g. GitHub). See the Nature Portfolio guidelines for submitting code & software for further information.

Data

Policy information about availability of data

All manuscripts must include a data availability statement. This statement should provide the following information, where applicable:

- Accession codes, unique identifiers, or web links for publicly available datasets
- A description of any restrictions on data availability
- For clinical datasets or third party data, please ensure that the statement adheres to our policy

All data to support the findings in this study are presented in the figures and can be made available upon reasonable request to the corresponding author. There are restrictions on the identity of tissue sources only.

Field-specific reporting

Please select the one below that is the best fit for your research. If you are not sure, read the appropriate sections before making your selection.

☒ Life sciences ☐ Behavioural & social sciences ☐ Ecological, evolutionary & environmental sciences

For a reference copy of the document with all sections, see nature.com/documents/nr-reporting-summary-flat.pdf

Life sciences study design

All studies must disclose on these points even when the disclosure is negative.

Sample size	Sample sizes were based on previous in vitro experiments conducted in the lab, and published studies of drug screening. Some experiments were limited due to tissue availability.
Data exclusions	No data were excluded from the analysis.
Replication	Experiments were repeated in cells/tissue from at least 3 different individuals. All attempts at replication were successful.
Randomization	Randomization was not performed in this study as it was not applicable to the experiments involved. All cases tested were subject to the same in vitro conditions.
Blinding	Analysis of immunofluorescence was automated by high-throughput imaging and analysis software. Blinding was conducted during cytometric bead array experiments, and histological staining.

Reporting for specific materials, systems and methods

We require information from authors about some types of materials, experimental systems and methods used in many studies. Here, indicate whether each material, system or method listed is relevant to your study. If you are not sure if a list item applies to your research, read the appropriate section before selecting a response.

Materials & experimental systems

n/a	Involved in the study	
☐	☒	Antibodies
☒	☐	Eukaryotic cell lines
☒	☐	Palaeontology and archaeology
☒	☐	Animals and other organisms
☒	☐	Human research participants
☒	☐	Clinical data
☒	☐	Dual use research of concern

Methods

n/a	Involved in the study	
☒	☐	ChIP-seq
☐	☒	Flow cytometry
☒	☐	MRI-based neuroimaging

Antibodies

Antibodies used

β-actin (AC-15) Rabbit Abcam ab6276 - - 1:2000 -
Akt (40D4) Mouse Cell Signalling 2920 - - 1:2000 -
pAkt (poly) Rabbit Cell Signalling 9271 - - 1:1000 -
cJun (poly) Rabbit Santa Cruz sc-1694 1:500 - - -
EEA-1 (poly) Rabbit Abcam ab2900 1:10000 - - -
EGR-1 (poly) Rabbit Santa Cruz sc-198 1:500 - - -
EGR-1 (15F7) Rabbit Cell Signalling 4153 - - 1:500 -
ERK1/2 (L34F12) Mouse Cell Signalling 4696 - - 1:500 -
pERK1/2 (D13.14.4E) Rabbit Cell Signalling 4370 - - 1:500 -

pERK1/2 (poly) Rabbit Cell Signalling 9101 1:1000 - - -
GAPDH (Abcam 9484) Mouse Abcam ab9484 - - 1:2000 -
IgG2a-κ control (G155-178) Mouse BD Pharmingen 555571 - - - 1:20
IL-6 (poly) Goat R&D AF-206 1:2500 - - -
Ki67 (MIB-1) Mouse Dako M7240 1:500 - - -
LAMP-1 (H4A3) Mouse DSHB AB_2296838
1:500 - - -
MCP-1 (poly) Rabbit Abcam ab9669 1:500 - - -
NF-κB p65 (poly) Rabbit Santa Cruz sc-372 1:500 - - -
NF-κB p65 (F-6) Mouse Santa Cruz sc-8008 1:500 - - -
PDGFRβ (BR7212) Mouse BioRad 7460-3104 1:500 - - 1:500
PDGFRβ (Y92) Rabbit Abcam ab32570 1:500 1:100 - -
PDGFRβ (poly) Goat R&D AF385 1:1000 - 1:1000 -
PDGFRβ-PE (28D4) Mouse BD Pharmingen 558820 - - - 1:20
pPDGFRβ (C63G6) Rabbit Cell Signalling 4549 - - 1:1000 -
Rab5 (poly) Rabbit Abcam ab18211 1:500 - - -
Rab7 (EPR7589) Rabbit Abcam ab137029 1:500 - - -
SMAD2/3 (C-8) Mouse Santa Cruz sc-133098 1:500 - - -
STAT1 (D1K9Y) Rabbit Cell Signalling 14994 1:500 - - -
STAT3 (124H6) Mouse Cell Signalling 9139 1:500 - - -
Biotinylated UEA lectin - Vector Labs B-1065 - 1:1000 - -
Anti-mouse Alexa 488 Goat Life Tech A11001 1:500
Anti-mouse Alexa 594 Goat Life Tech A11005 1:500
Anti-mouse Alexa 647 Goat Life Tech A21235 1:500
Anti-mouse Alexa 488 Donkey Life Tech A21202 1:500
Anti-rabbit Alexa 488 Goat Life Tech A11008 1:500
Anti-rabbit Alexa 594 Goat Life Tech A11012 1:500
Anti-rabbit Alexa 647 Goat Life Tech A27040 1:500
Anti-rabbit Alexa 594 Donkey Life Tech A21207 1:500
Anti-rabbit Alexa 647 Donkey Life Tech A31573 -
Anti-goat Alexa 488 Donkey Life Tech A11055 1:500
Anti-goat Alexa 647 Donkey Life Tech A21447 1:500
Anti-mouse IRDye-680LT Goat LiCOR 926-68020 -
Anti-rabbit IRDye-800CW Goat LiCOR 926-32211 -
Anti-rabbit IRDye-800CW Donkey LiCOR 925-32213 -
Anti-goat IRDye-680LT Donkey LiCOR 926-68024 -
Streptavidin-Cy5 - Jackson IR 016-160-074 -

Validation

Antibodies used in this study have been either validated by the manufacturer, by siRNA knockdown or western blot.

Flow Cytometry

Plots

Confirm that:

☒ The axis labels state the marker and fluorochrome used (e.g. CD4-FITC).

☒ The axis scales are clearly visible. Include numbers along axes only for bottom left plot of group (a 'group' is an analysis of identical markers).

☐ All plots are contour plots with outliers or pseudocolor plots.

☒ A numerical value for number of cells or percentage (with statistics) is provided.

Methodology

Sample preparation

For cell surface labelling, primary human brain pericytes were brought into suspension with Accutase, then immediately stained with PDGFRB-PE in FACS buffer (1 mM EDTA, 1 % FBS, in PBS). Samples were washed and acquired. For total PDGFRB staining, samples were brought into suspension with Accutase, then fixed in 4 % PFA for 15 minutes. Samples were washed and permeabilised in PBS with 0.1 % Triton X-100 (v/v) and stained with mouse anti-PDGFRB overnight at 4 degrees in FACS buffer. Samples were washed and goat anti-mouse 488 added for 3 h at room temperature, washed, and acquired.

Instrument

BD Accuri C6

Software

FlowJo v7.6.5

Cell population abundance

Median fluorescence intensity of PDGFRB signals was quantified, rather than percentages.

Gating strategy

For live cells, cells were gated on FSC and SSC, multiplets were excluded based on FSC-A v FSC-H. For fixed cells, cells were gated on FSC and SSC, and NucRed inclusion, multiplets were excluded based on FSC-A v FSC-H.

☒ Tick this box to confirm that a figure exemplifying the gating strategy is provided in the Supplementary Information.
